# Deep tissue analysis of distal aqueous drainage structures and contractile features

**DOI:** 10.1038/s41598-017-16897-y

**Published:** 2017-12-06

**Authors:** Jose M. Gonzalez, Minhee K. Ko, Young-Kwon Hong, Robert Weigert, James C. H. Tan

**Affiliations:** 1 Doheny Eye Institute and Department of Ophthalmology, David Geffen School of Medicine at UCLA, Los Angeles, CA USA; 2 Department of Surgery, Department of Biochemistry and Molecular Biology, Norris Comprehensive Cancer Center, Keck School of Medicine, University of Southern California, Los Angeles, California, USA; 3 Intracellular Membrane Trafficking Section, National Institute of Dental and Craniofacial Research, National Institutes of Health, Bethesda, Maryland USA

## Abstract

Outflow resistance in the aqueous drainage tract distal to trabecular meshwork is potentially an important determinant of intraocular pressure and success of trabecular bypass glaucoma surgeries. It is unclear how distal resistance is modulated. We sought to establish: (a) multimodal 2-photon deep tissue imaging and 3-dimensional analysis of the distal aqueous drainage tract (DT) in transgenic mice *in vivo* and *ex vivo*; (b) criteria for distinguishing the DT from blood and lymphatic vessels; and (c) presence of a DT wall organization capable of contractility. DT lumen appeared as scleral collagen second harmonic generation signal voids that could be traced back to Schlemm’s canal. DT endothelium was Prox1-positive, CD31-positive and LYVE-1-negative, bearing a different molecular signature from blood and true lymphatic vessels. DT walls showed prominent filamentous actin (F-actin) labeling reflecting cells in a contracted state. F-actin co-localized with mesenchymal smooth muscle epitopes of alpha-smooth muscle actin, caldesmon and calponin, which localized adjacent and external to the endothelium. Our findings support a DT wall organization resembling that of blood vessels. This reflects a capacity to contract and support dynamic alteration of DT caliber and resistance analogous to the role of blood vessel tone in regulating blood flow.

## Introduction

Glaucoma is the leading cause of irreversible blindness worldwide and intraocular pressure (IOP) is a major risk factor for the disease. The trabecular meshwork (TM) is a key resistance point in the aqueous humor drainage tract and important determinant of IOP. But drainage pathways downstream to the TM may be nearly as important as up to half of total outflow resistance has been attributed to the intrascleral distal aqueous drainage tract (DT)^1–10^. The DT comprises Schlemm’s canal (SC), collector channels (CC), intrascleral plexus (ISP) and aqueous veins connecting to episcleral veins.

The clinical importance of the DT has been brought into fresh focus by newer glaucoma surgeries that bypass TM resistance by incising the TM or inserting drainage stents through it. Following these procedures, IOP may be lowered but generally not to the anticipated level of episcleral venous pressure (EVP). This raises a suspicion that significant outflow resistance must remain in the DT after the TM is bypassed.

The extent to which the DT is able to modulate outflow resistance is unknown, although a capacity to contract has been suggested^8,11,12^. If so it may be that the DT has a contractile apparatus analogous to blood vessels lying downstream from it. Capacity of blood vessels to contract permits alteration of vessel caliber and modulation of flow resistance. Perhaps a similar mechanism is relevant to the DT apparatus lying upstream and in continuity with the episcleral veins. If such a mechanism applies to distal aqueous drainage it would suggest that resistance here is neither static nor passive, but rather that it is dynamic and regulated.

We postulate that the walls of the DT lying beyond the TM incorporate contractile cells with a smooth muscle identity in an organization mimicking conventional blood vessels. In blood vessels, smooth muscle forms a middle coat (tunica media) of the wall. It is sandwiched between an inner endothelial layer next to the lumen and a strong outer adventitial coat of collagen structural extracellular matrix (ECM). Episcleral veins that are conventional blood vessels into which the DT empties would have such an organization. More proximally, ciliary muscle (CM) that participates in uveoscleral aqueous drainage is itself smooth muscle.

We conducted tissue-based studies to characterize the fine structure and aspects of the molecular identity of the DT and explore whether it has contractile features. We studied this in mice as its aqueous drainage tract is similar to that of primates in structure and function^13–19^. We used transgenic fluorescent reporter mice expressing specific endothelial tags to improve identification of the DT. The DT has a unique partial lymphatic identity^20–23^, with endothelium here expressing Prospero homeobox protein 1 (Prox1) but not lymphatic vessel endothelial hyaluronal receptor-1 (LYVE-1) or other lymphatic endothelial markers typically expressed in true lymphatic vessels^22,24–26^. While blood vessel endothelium and DT endothelium share in common expression of typical endothelial markers such as vascular endothelial growth factor receptor 2 (VEGFR2), platelet endothelial cell adhesion molecule 1 (PECAM-1 or CD31) and vascular endothelial cadherin (VE-cadherin), blood vessels do not express lymphatic endothelial markers such as Prox1^27–30^.

We used trans-scleral multimodal 2-photon (2 P) imaging combining 2 P excitation fluorescence (TPEF) and second harmonic generation (SHG)^31–45^ *in vivo* and *ex vivo*. This approach recognizes the DT structural and functional continuity with the systemic circulation and importance of maintaining this context. It was necessary to confirm the structures under study were DT channels rather than blood or lymph-carrying vessels, which also traverse the limbus. This led us to establish criteria to confirm that DT structures (a) were continuous with SC and (b) showed the unique endothelial molecular signature expected of the system.

## Results

### Tissue-based identification of distal aqueous drainage tract structures

**Figure 1:**
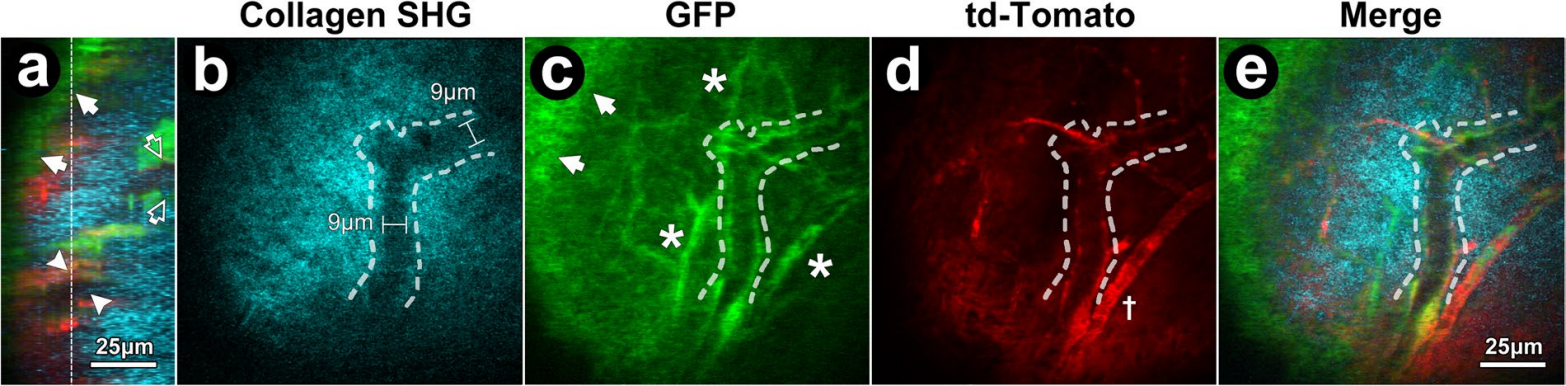
*In vivo* trans-scleral 2-photon imaging of distal aqueous drainage channels and cells of GFP/mTomato mice. (**a**) Orthogonal image reconstruction shows mosaic td-Tomato (red) and GFP (green) expression in the conjunctiva (*arrows*) and deep to the ocular surface especially in the distal aqueous drainage tract. Deeper signals were associated with a lumen (SHG (cyan) signal voids) of an intrascleral plexus (ISP; *arrowheads*) and collector channel opening into Schlemm’s canal (SC; *open arrows*). *Vertical dashed line:* scleral location of ISP in optical sections (**b**–**e**). (**b**) Void in SHG signal (cyan; sclera) represents ISP lumen and non-collagenous cell wall with a width of 9 μm in a location deep to the ocular surface. (**c**) GFP expression in ISP wall and other intrascleral vessels (*asterisks*) deep to conjunctiva (*arrows*). (**d**) Membrane-targeted td-Tomato (red) expression in cells of ISP wall (*hashed outline*) and other vessels (*cross*). (**e**) Merge of collagen SHG, GFP, and td-Tomato signals showing ISP (*Curved hashed outline*).

**Figure 2:**
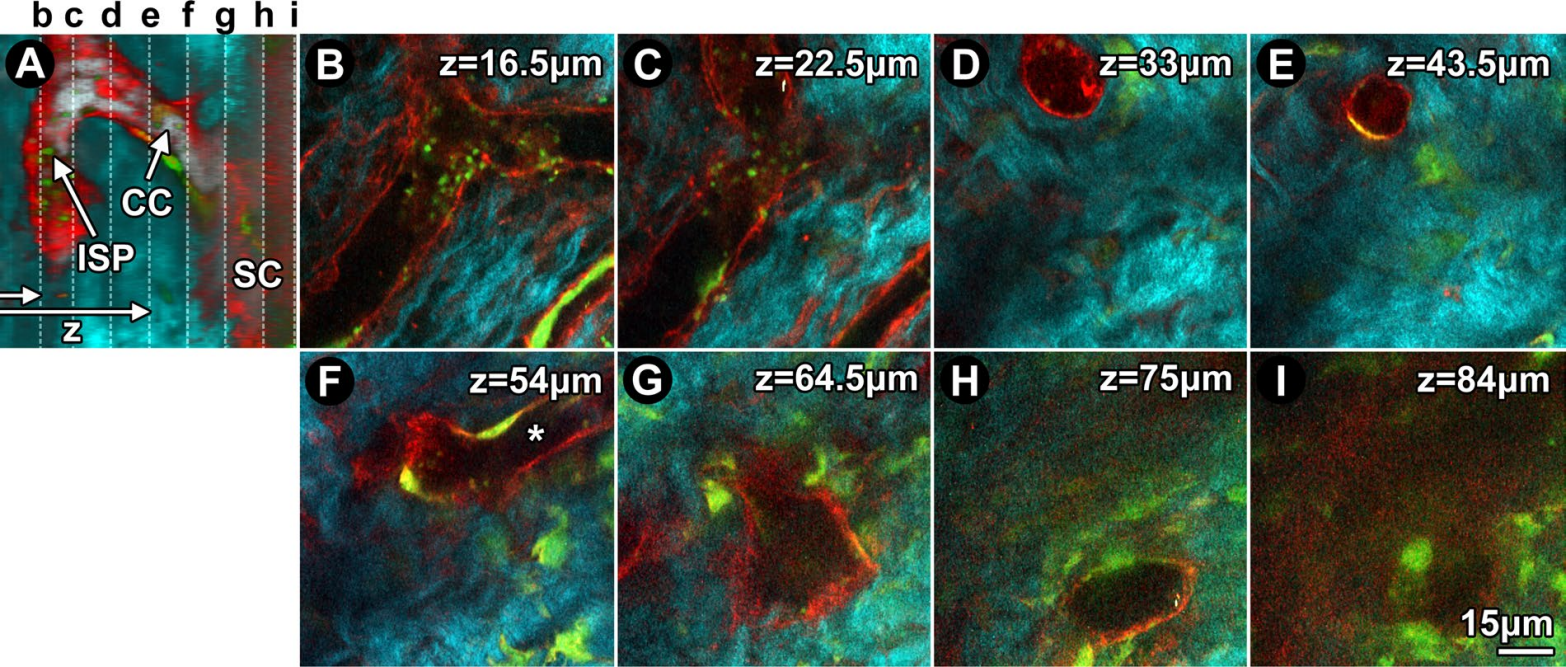
*Ex vivo* trans-scleral 2-photon imaging of structure and cells in distal aqueous drainage tract of GFP/mTomato mice. (**A**) Orthogonal image reconstruction showing td-Tomato (red) and GFP (green) lining an intrascleral plexus (ISP), collector channel (CC) and outer wall of Schlemm’s canal (SC) within scleral collagen (SHG; cyan). *Vertical dashed lines*: serial depths (z) of optical slices shown in b-i. *Horizontal arrows*: laser path. (**B**) Branching ISP lumen located z = 16.5 μm deep to the conjunctival epithelium. (**C**) At z = 22.5 μm, ISP connection to a CC is evident. (**D**) At z = 33 μm, the vertically orientated CC is clearly separate from ISP. (**E**) At 43.5 μm, the CC maintained its course but narrows. (**F**) At z = 54 μm, the CC merges with a different, deeper ISP (asterisk). (**G**) At 64.5 μm, the CC widens as it approaches SC. (**H**) At z = 75 μm, the CC opens into SC at a collector channel opening (CCO). (**I**) View inside SC wherein a mosaic of GFP and td-Tomato-positive cells populate the outer wall.

We first identified intrascleral DT structures and confirmed they were traceable to SC. Mouse sclera is thin and its DT apparatus is near the ocular surface, making it amenable to trans-scleral visualization by deep tissue imaging techniques. We used TPEF to view autofluorescence and transgenic reporter fluorescence, and SHG, a label-free 2 P technique to view structural collagen^32,37,41,45^. Imaging of hybrid cell membrane-localized td-Tomato (mTomato or mT) and cytosolic green fluorescent protein (GFP) co-expressing (mT/GFP) transgenic reporter mice revealed an interconnected network of intrascleral channels. The channels were evident as voids in the scleral collagen SHG signal deep to the ocular surface, as shown in Fig. 1 (*in vivo*) and 2 (*ex vivo*). The channel voids of the DT could be seen extending distally from SC toward the ocular surface (Figs 1a and 2A). Thus it was possible to trace DT channels nearer the ocular surface such as ISPs and CCs back to SC in 2 P serial image sections.

The hybrid transgenic mosaic expression profile of GFP (cytosolic) and td-Tomato (membrane) indicated the location of cells within the collagenous structural ECM and walls of channels and vessels in the sclera. The collagenous structural ECM was revealed by SHG to an extent that wavy collagen bundles could be resolved (e.g., Fig. 2B–E). SHG signal voids indicating absence of structural collagen correlated with lumen and cell complexes in the walls of intrascleral channels and vessels. The location of cell complexes at the edge of SHG signal voids (Fig. 1b) was revealed by GFP (Fig. 2C) and/or td-Tomato (Fig. 2D) mosaic fluorescence typical of the GFP/mT hybrid mice we used^46^. Thus simultaneous visualization of lumen SHG signal voids and wall cell-based fluorescence allowed delineation of the configuration of intrascleral channels and vessels (Fig. 2A–I).

Branching ISPs in optical sections were orientated in a plane parallel to the ocular surface (Fig. 2B–C). CCs, having a more radial orientation to the ocular surface, appeared in cross-section as rounder structures (Fig. 2D,E). The CCs were connections between ISPs (Fig. 2F,G) and SC (via collector channel openings [CCO]; Fig. 2H,I).

By confirming that intrascleral channels had their origins in SC we were able to verify they were a part of the distal aqueous drainage tract.

### Isosurface mapping and 3D reconstruction of distal aqueous drainage tract

Having verified that intrascleral structures under study were part of the DT, we used their luminal SHG signal voids to map and 3-dimensionally reconstruct the intrascleral course of the tract using software-based techniques. This would permit better characterization of the 3-dimensionally complex DT and its relationships with endothelial and contractile markers.

**Figure 3:**
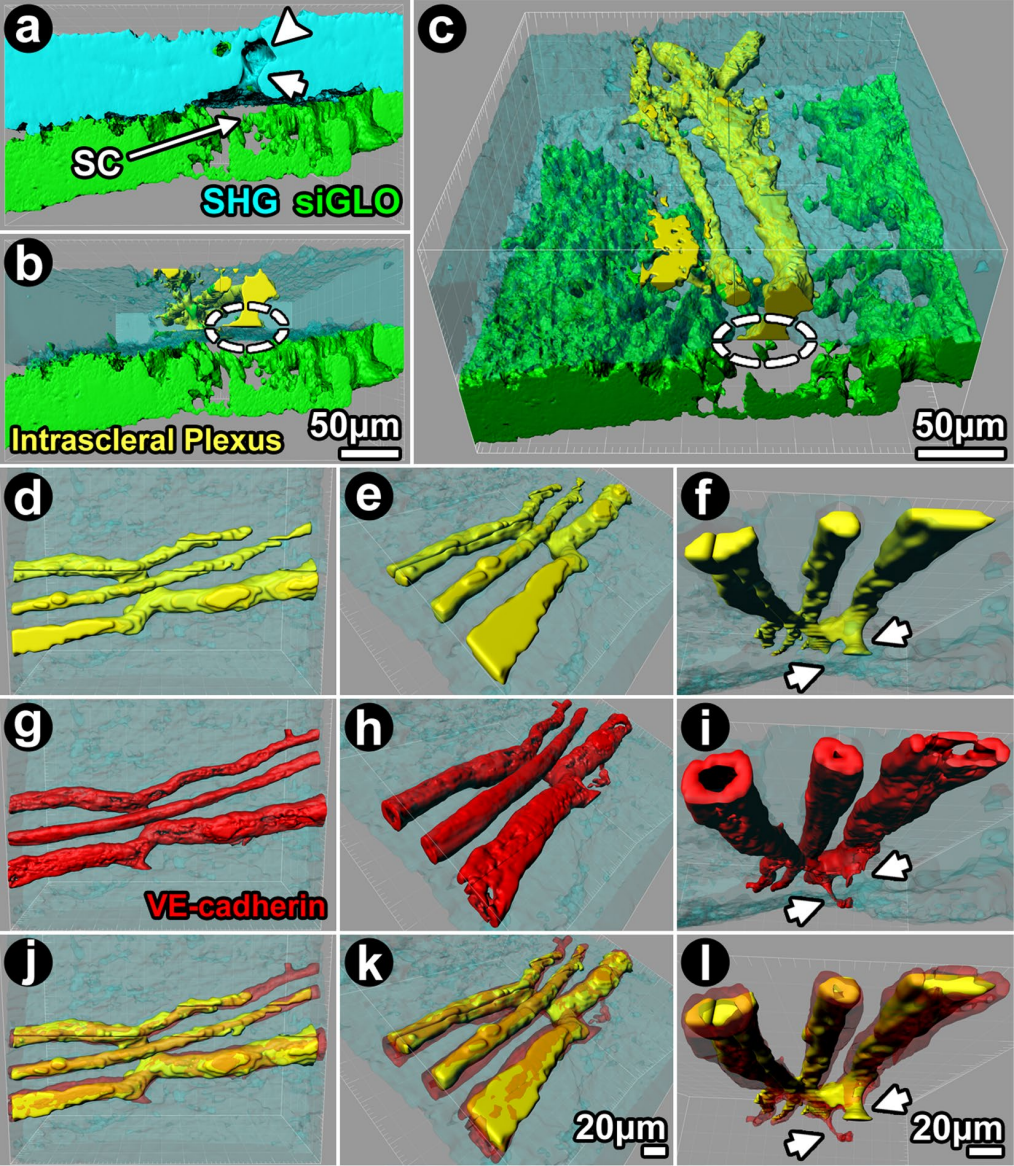
Isosurface mapping and 3-dimensional reconstruction of distal aqueous drainage tract. Reconstruction was based on 2-photon serial optical sections, merging signals from scleral collagen SHG (cyan; **a**), SHG signal voids (**b**) and siGLO-related intracellular fluorescence (green; **c**) in eyes from Balb/c (**a**–**c**) and VE-cadherin-td-Tomato (**d**–**l**) mice. siGLO was taken up by trabecular meshwork (TM) and intrascleral plexus (ISP) endothelium after mouse intracameral perfusion. Isosurface-mapped negative imprints of SHG signal voids were used to reconstruct collector channels (CC) and ISPs and assigned as yellow. Schlemm’s canal (SC) was a slit-like opening between scleral SHG and TM siGLO signals. Continuation of SC openings into CCs (yellow) were seen. (**a**) Orthogonal reconstruction of SHG signal voids showing lumen of ISP and CC connecting to SC. CC ostium (CCO) and CC (*arrow*) connect SC to an ISP (*arrowhead*). (**b**) Sclera (SHG) was rendered transparent to reveal the relationships of ISPs with CC (yellow), collector channel openings (CCO; *dashed circle*), SC (gap) and TM (green) in 3D space. (**c**) The 3D model is rotated, showing a trans-scleral view from the ocular surface of interconnected ISPs, CC, CCO (*dashed circle*), SC (gap) and TM (green). (**d-i**) Isosurface-mapped, 3D-reconstructed ISPs derived from (*i*) negative imprints of SHG signal voids (**d**–**f**); yellow; representing ISP lumen) and (*ii*) VE-cadherin-expressing endothelium (**g**–**i**; red) of a *VE-cadherin-td-Tomato* mouse. The same branching ISPs were rotated to provide different views within sclera (cyan; rendered transparent). (**j**–**l**) merge of (**d**–**f**) (lumen) and (**g**–**i**) (endothelium) showing endothelium wrapped around ISP lumen. *Arrows:* ISP connecting to CC (**f**).

siGLO delivered intracamerally was used to localize cells along the DT of wild-type Balb/c and C57BL/6 mouse eyes. siGLO was taken up primarily by TM cells and ISP endothelium and used to map the location of TM relative to the adjacent sclera (SHG; cyan) and distal tract (Fig. 3a showing SHG voids and ISP (green); yellow in Fig. 3b–c). With the scleral SHG signal rendered transparent (Fig. 3b,c), the complex 3D configuration of the DT and its relationships with surrounding sclera and proximal TM were evident. Reconstructions showed ISP branching, interconnection and coursing at different scleral depths, as was suggested by Fig. 2B and F. The trans-scleral vertical connection of ISPs, CC and SC in the trabecular outflow pathway was also evident.

SHG signal voids of the DT were further investigated for the presence of VE-cadherin using TPEF imaging of VE-cadherin-td-Tomato expressing mouse eyes (Fig. 3d–i). VE-cadherin is an archetypal endothelial marker and its expression signified presence of endothelium (Fig. 3g–i). SHG signal voids (Fig. 3d–f) represented mainly lumen. By rendering the scleral SHG signal transparent, branching ISPs and their relationship with a CC could be viewed from different angles in 3D space. VE-cadherin-positive endothelium was arranged around a lumen (e.g., Fig. 3i) corresponding to SHG signal voids (Fig. 3f). There was some overlap between the endothelial td-Tomato signal and SHG signal void, however (Fig. 3l), as the SHG signal void also represented non-collagenous elements (e.g., cells, ECM) in the ISP wall. These findings provided validation for our use of SHG signal voids as surrogates to locate structures of the DT.

### Characterizing endothelium of the distal aqueous drainage tract *in situ*

We further characterized the DT by profiling endothelial markers to verify the presence of a unique endothelial molecular signature expected of the system.

**Figure 4:**
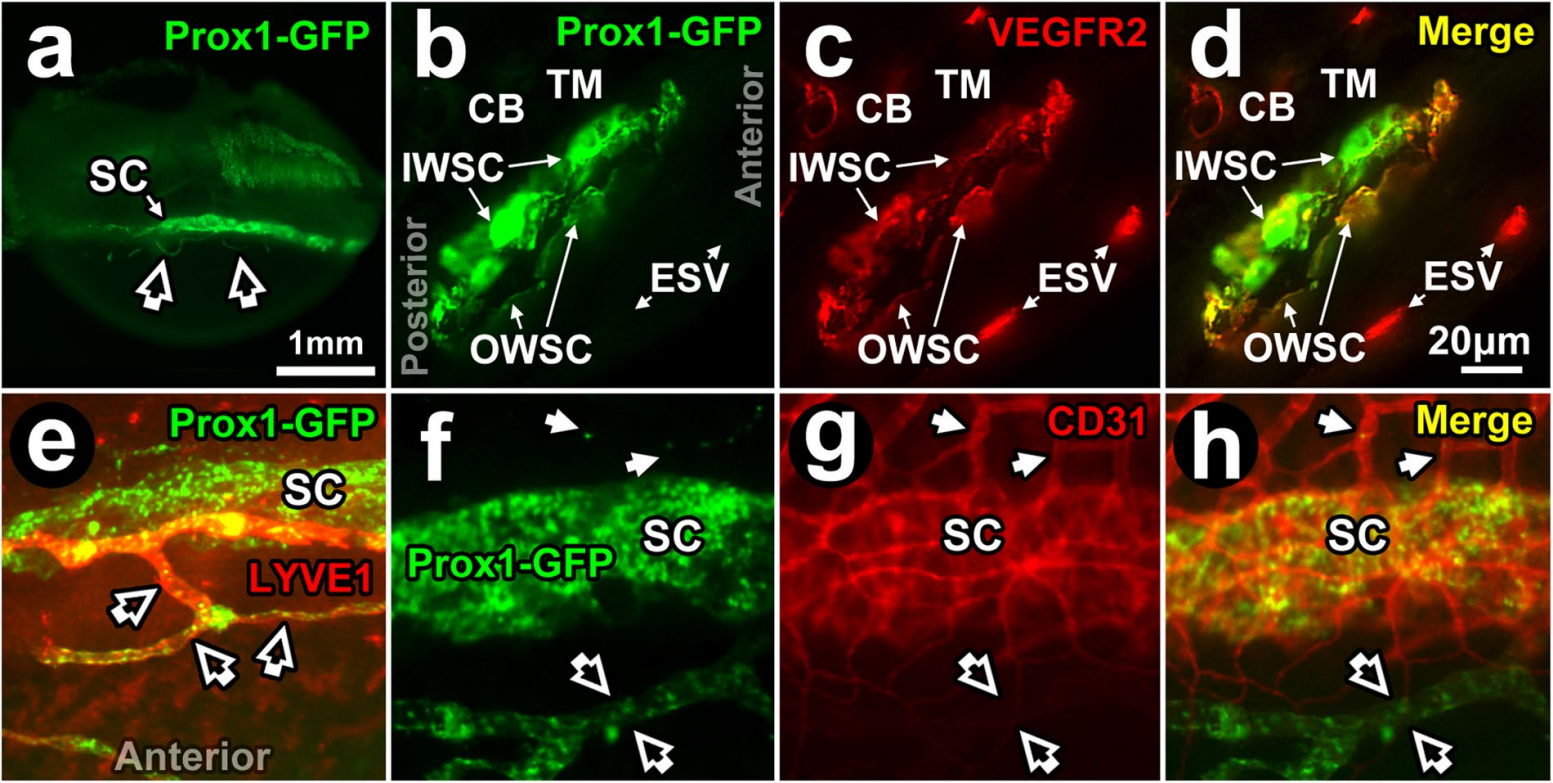
Prox1-GFP expression in Schlemm’s canal. (**a**) Prox1-GFP expression (bright green band) in *Prox1-GFP* mouse Schlemm’s canal (SC) seen by imaging the external limbus *ex vivo* (external eye surface faces reader). The cornea lies inferiorly. *Open arrows:* anterior Prox1-GFP-positive anterior projections from limbal lymphatics (see later Fig. 5). (**b**–**d**) Fluorescence microscopy of a frozen section (10 μm thickness) of Prox1-GFP mouse angle tissue after labeling with anti-VEGFR2 antibodies. (**b**) At higher magnification, Prox1-GFP expression was more intense in the inner wall (IWSC) than outer wall of SC (OWSC). Prox1-GFP expression was not seen in the trabecular meshwork (TM), ciliary body (CB), and episcleral vessels (ESV). (**c**) VEGFR2 staining intensity was similar in IWSC, OWSC, CB blood vessels and episcleral veins (ESV). (**d**) A merged image shows Prox1-GFP co-localization with VEGFR2 in IWSC and OWSC but not ESV. (**e**) Merged image showing Prox1-GFP and LYVE-1 expression. Prox1-GFP is expressed in both SC (green) and lymphatic vessels (*open arrows*) but only lymphatic vessels express LYVE-1 that co-localizes with Prox1 (orange). (**f**–**h**) SC, lymphatic vessels (*open arrows*) and blood vessels (*closed arrows*) at the limbus. (**f**) Prox1 (green) is expressed in SC and lymphatic vessels but not blood vessels. (**g**) CD31 (red) is expressed in SC and blood vessels but is low in lymphatic vessels. (**h**) Merged image shows Prox1 and CD31 co-localization in SC (orange) but not in blood vessels (red) or lymphatic vessels (green).

**Figure 5:**
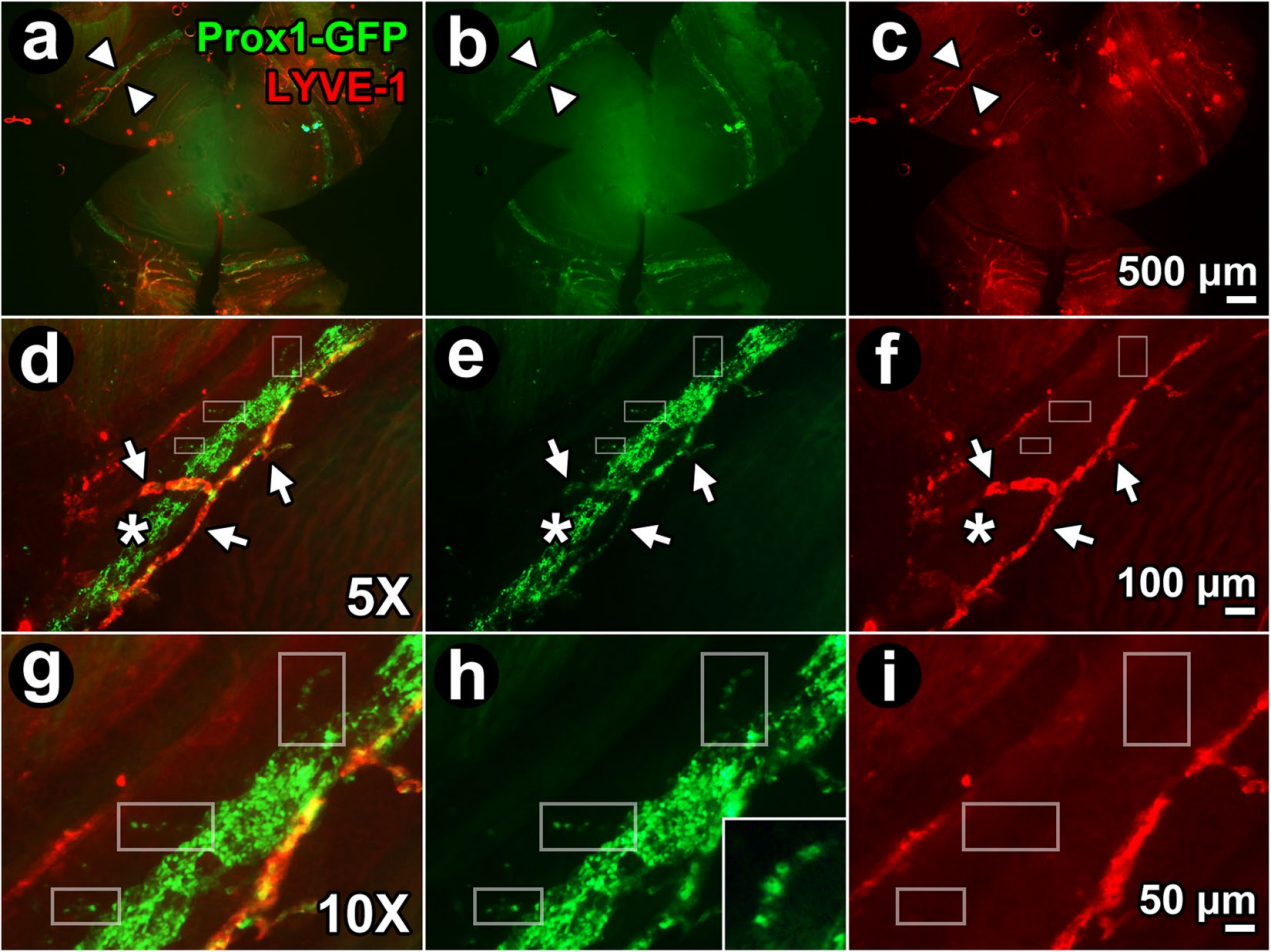
Expression of Prox1-GFP expression in collector channels. (**a**–**c**) Whole mount (external corneal surface faces reader) of *Prox1-GFP* mouse eye labeled with anti-LYVE-1 antibodies. (**a**) Merged image shows LYVE-1-positive, Prox1-positive (orange/yellow with co-localization) limbal lymphatic vessels close to Schlemm’s canal (SC; green). SC was a ~125 μm wide circumferential band showing only Prox1-GFP-positivity. *Arrowheads:* region to be magnified in subsequent panels, (**b**–**i**). (**b,e,h**) Green channel image showing Prox1-GFP-positive structures, representing both distal aqueous drainage tract and limbal lymphatics. (**c,f,i**) Red channel image showing LYVE-1-positive structures only, representing limbal lymphatics but not SC or distal aqueous drainage tract. (**d**–**f** and **g**–**i**): 5X and 10X magnification respectively of region indicated by arrowheads in panel (a–c). *Arrows:* LYVE-1-positive, Prox1-positive limbal lymphatic vessels that are orange/yellow from co-localization. Prox1-GFP and LYVE-1 co-localize (orange/yellow) in limbal lymphatic vessels, but CC (boxes in g,h; inset in h; discontinuous expression) and SC express Prox1-GFP only.

Prox1 is strongly expressed in the endothelium of SC^24,25,28,47–49^. Prox1 is also expressed in the DT distal to SC^42^ but past reports define this less clearly. We sought to localize regions of Prox1-positive endothelium in the DT of *Prox1-GFP* reporter mice by TPEF (Figs 4 and 5). Prominent Prox1 expression was seen in the SC endothelium (Fig. 4a,b, green). Here, Prox1 co-localized (Fig. 4d, yellow) with VEGFR2 (Fig. 4c, red), an endothelial marker. Prox1 expression was more prominent in the inner wall (IWSC) than in the outer wall (OWSC) of SC (Fig. 4b). VEGFR2 but not Prox1-GFP expression was seen in blood vessels such as episcleral veins or ciliary body vasculature (Fig. 4d).

True lymphatic vessels expressed Prox1-GFP as well (Fig. 4e). In Prox1-GFP mice, focal regions of hyperfluorescence with regular periodicity representing Prox1-GFP expression within lymphatic valves were seen in lymphatic vessels but not in SC^25^. While Prox1-GFP was expressed in the endothelia of both SC and lymphatic vessels (Fig. 4e,f), only true lymphatic vessels expressed LYVE-1 (Fig. 4e). Blood vessels expressed CD31 but not Prox1 (red; Fig. 4g). CD31 expression was very low and appeared almost imperceptible in lymphatic vessels (Fig. 4g) but it was clearly expressed in SC (Fig. 4g) where it co-localized with Prox1 (Fig. 4h).

A fuller characterization and comparison of lymphatic vessels and DT channels was performed (Fig. 5). True lymphatic vessels at the limbus were identified by anti-LYVE-1 antibody labeling wherein the positive LYVE-1 signal co-localized with Prox1-GFP. Unlike lymphatic vessels, Prox1 co-localization with LYVE-1 was not seen in SC or CC. The pattern of Prox1-GFP expression in CC was patchy, appearing discontinuous and beaded. This simulated on a smaller scale the patchy Prox1-GFP expression seen in SC (Fig. 5d–f and g-i).

**Figure 6:**
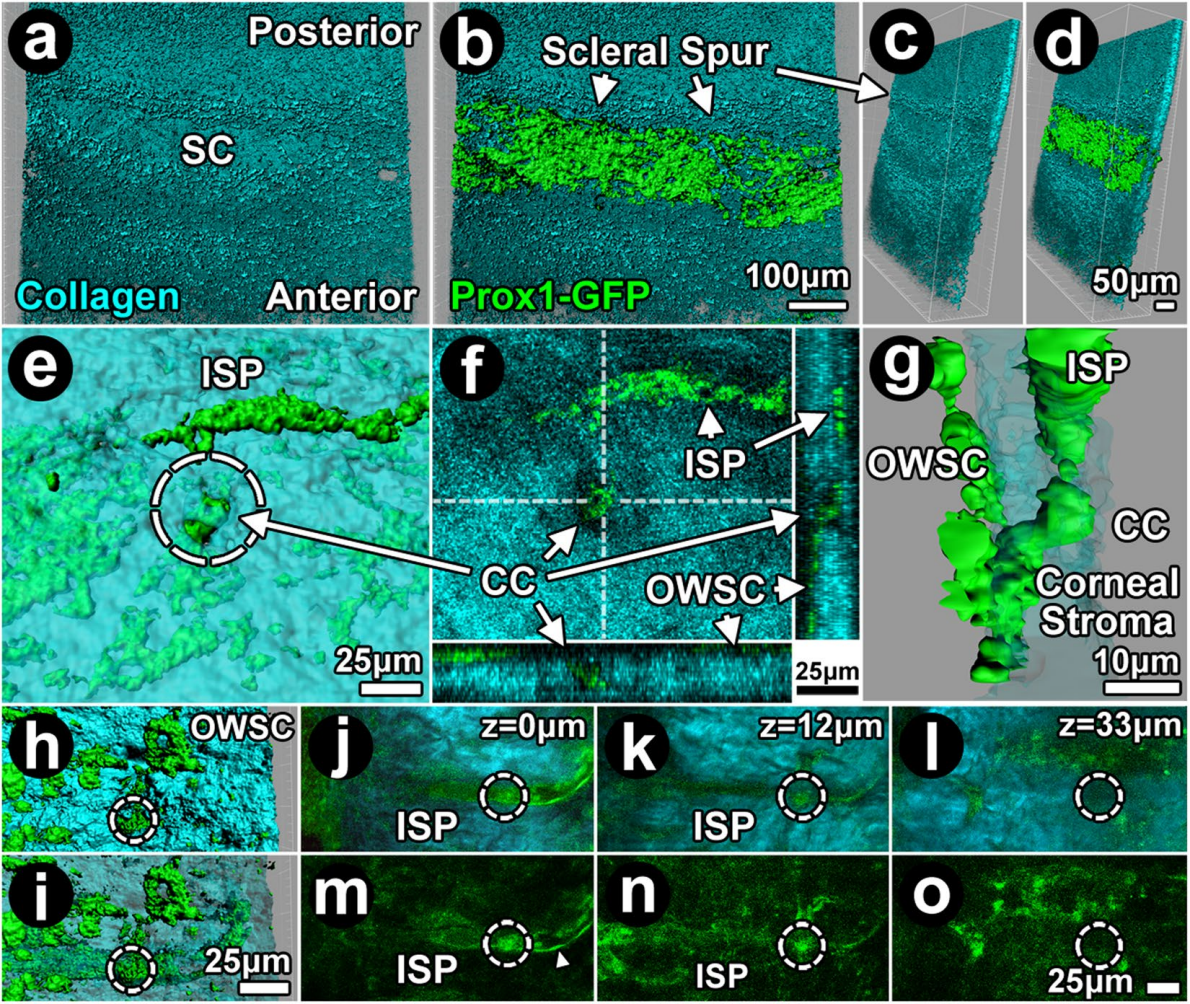
Continuous expression of Prox1-GFP from Schlemm’s canal to intrascleral plexus. (**a**–**d**) Isosurface mapping merging collagen second harmonic generation (SHG; cyan; **a** (en face), **c** (rotated)) and Prox1-GFP signals (green; **b** (en face), **d** (rotated)) revealed Prox1-GFP localization to a channel-shaped depression in sclera consistent with Schlemm’s canal (SC). A collagen ridge bordered the scleral depression posteriorly, consistent with a mouse equivalent of scleral spur. (**e**–**g**) Prox1-GFP expression in collector channel (CC; *arrows*; cross-sectional view) and intrascleral plexus (ISP) was continuous with scleral collagen SHG signal voids (*dashed circle*: CC lumen). This is illustrated by 2-photon (2 P) direct visualization (**f**), isosurface mapping (e) and 3D reconstruction (**g**). 3D reconstruction (**g**) showed CC continuous with the outer wall of Schlemm’s canal (OWSC) and ISP. (**h**–**i**) Isosurface mapping showed Prox1-GFP expression extending from the OWSC to ISP. (**h**) Patchy Prox1-GFP expression in OWSC and a collector channel (CC) opening (*dashed circle*). (**i**) The collagen SHG signal is rendered transparent, revealing the course of a CC (*dashed circle*) to an ISP more externally in the sclera. Both the CC and ISP express Prox1-GFP. (**j**–**o**) Serial 2 P optical sections through an ISP showed endothelial Prox1 expression in a fine, diaphanous layer (*arrowhead*) bordering the ISP lumen. Dashed circle indicates location of a CC. **(j**–**l**) are images merging collagen SHG and Prox1-GFP signals. (**m**–**o**) images showing Prox1-GFP expression only.

Endothelial Prox1-GFP expression was analyzed with reference to scleral collagen SHG signal voids and found to be present in the DT from SC to ISP (Fig. 6). In the inner sclera, isosurface mapping localized Prox1-GFP expression to a discrete broad, shallow trench in the scleral collagen SHG signal contour corresponding to SC (Fig. 6a–d). A posterior ridge of collagenous condensation bordering SC corresponded to the mouse equivalent of a scleral spur. Similar to SC, Prox1-GFP expression localized to the region of scleral SHG signal voids corresponding to the location of CC and ISP, as revealed by direct visualization (Fig. 6f,j–l,m–o), isosurface mapping (Figs 6e,h,i) and 3D reconstructions (Fig. 6g).

### Contractile cells with smooth muscle identity in distal aqueous drainage tract walls

To explore the possibility that the DT is contractile, we sought to locate contractile cells that may be associated with the tract and determine whether they possessed a smooth muscle identity. A similar organization is seen in blood vessels in which smooth muscle contractility plays important roles in modulating caliber and flow resistance.

Filamentous actin (F-actin), the polymerized, contractile form of actin, was labeled *in situ* to locate tissues in the DT that may be contractile. Fluorescence dye-conjugated phalloidin that allows stabilization and preferential detection of contractile states and stress fibers was used for labeling^44,50,51^.

**Figure 7:**
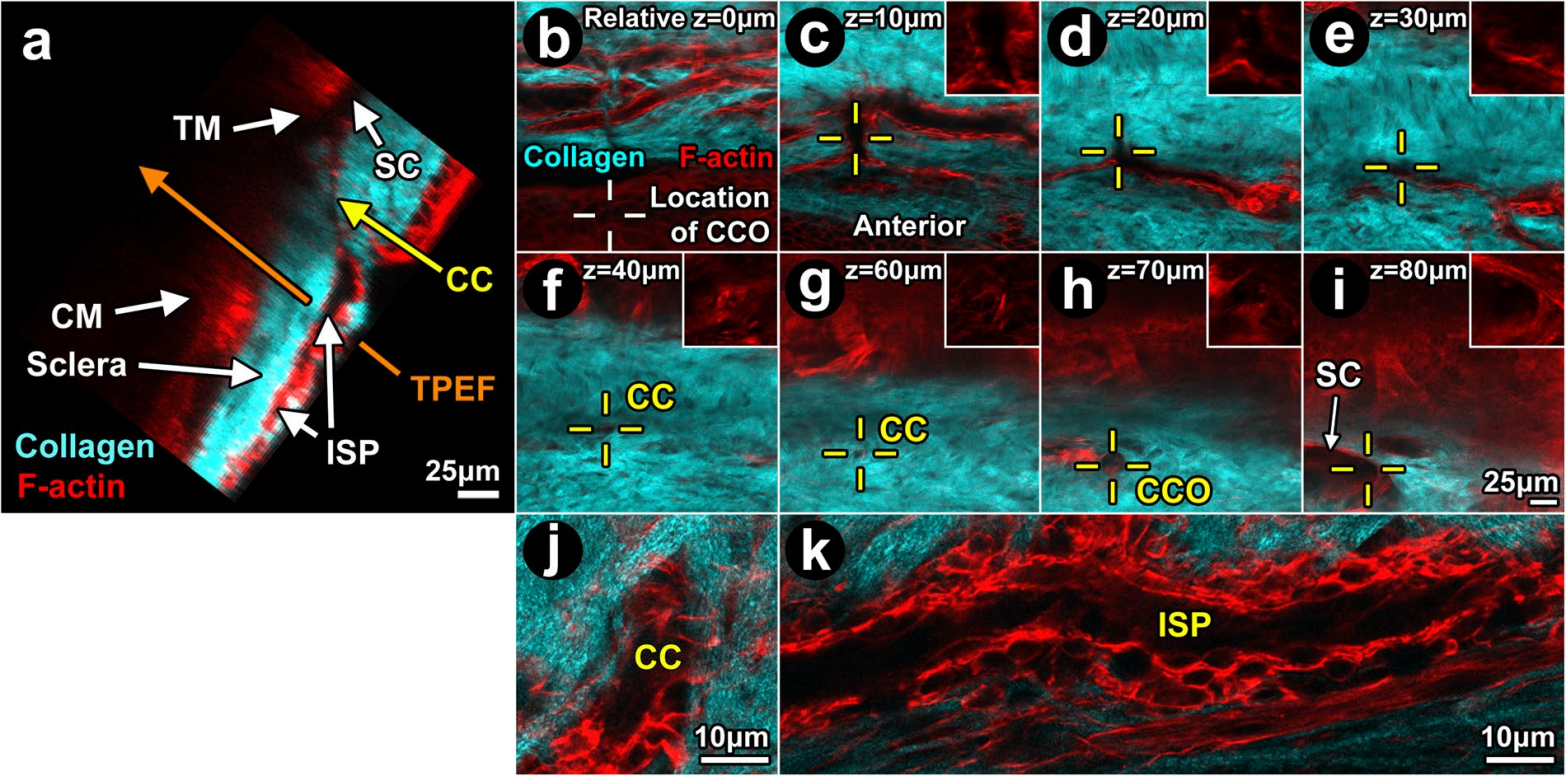
Filamentous actin in distal aqueous drainage tract walls. Orthogonal reconstruction (**a**) of serial optical sections (**b-i**) captured during *Balb/c* 2-photon trans-scleral imaging *ex vivo*. Eyes were labeled with Alexa-568-conjugated phalloidin for filamentous actin (F-actin; red). (**a**) Orthogonal image showing structures of ciliary muscle (CM; ~125 μm deep) and trabecular meshwork (TM) adjacent to Schlemm’s canal (SC). SC was continuous with a collector channel (CC) within scleral collagen (second harmonic generation (SHG); cyan). The CC connected with an intrascleral plexus (ISP). Optical sections captured progressively deeper in the sclera (**b-i**) showed the following: (**b**) prominent F-actin-positive labeling bordered the ISP lumen (SHG signal void); *White crosshairs:* location of CC ostium 70–80 μm deeper where ISP eventually connected to SC; (**c**) ISPs interconnect; *Yellow crosshairs:* bridging vessel connects with collector channel; (**d**) Relationship between CC and ISP at a deeper location; (**e**) Evidence of this relationship persisted; (**f-g**) CC course deeper into sclera (yellow crosshairs; relative depth 40–60 μm); (**h**): CC opening (CCO) into SC (relative depth 70 μm); (**i**) Deeper, SC was a fuller void in the collagen SHG signal, seen with reference to the CCO. The SHG signal (cyan) reveals collagenous bundles in sclera; (**j-k**): High magnification images of CC (**j**) and ISP (**k**) showing cytosolic F-actin outlining nuclei (*dark ovals*) and revealing a multilayered cellular wall organization surrounding lumen (dark central region) within SHG signal voids. The lumen comprise but a portion of the cross-sectional area of each intrascleral channel, with cells filling the rest of the area as part of the channel wall.

**Figure 8:**
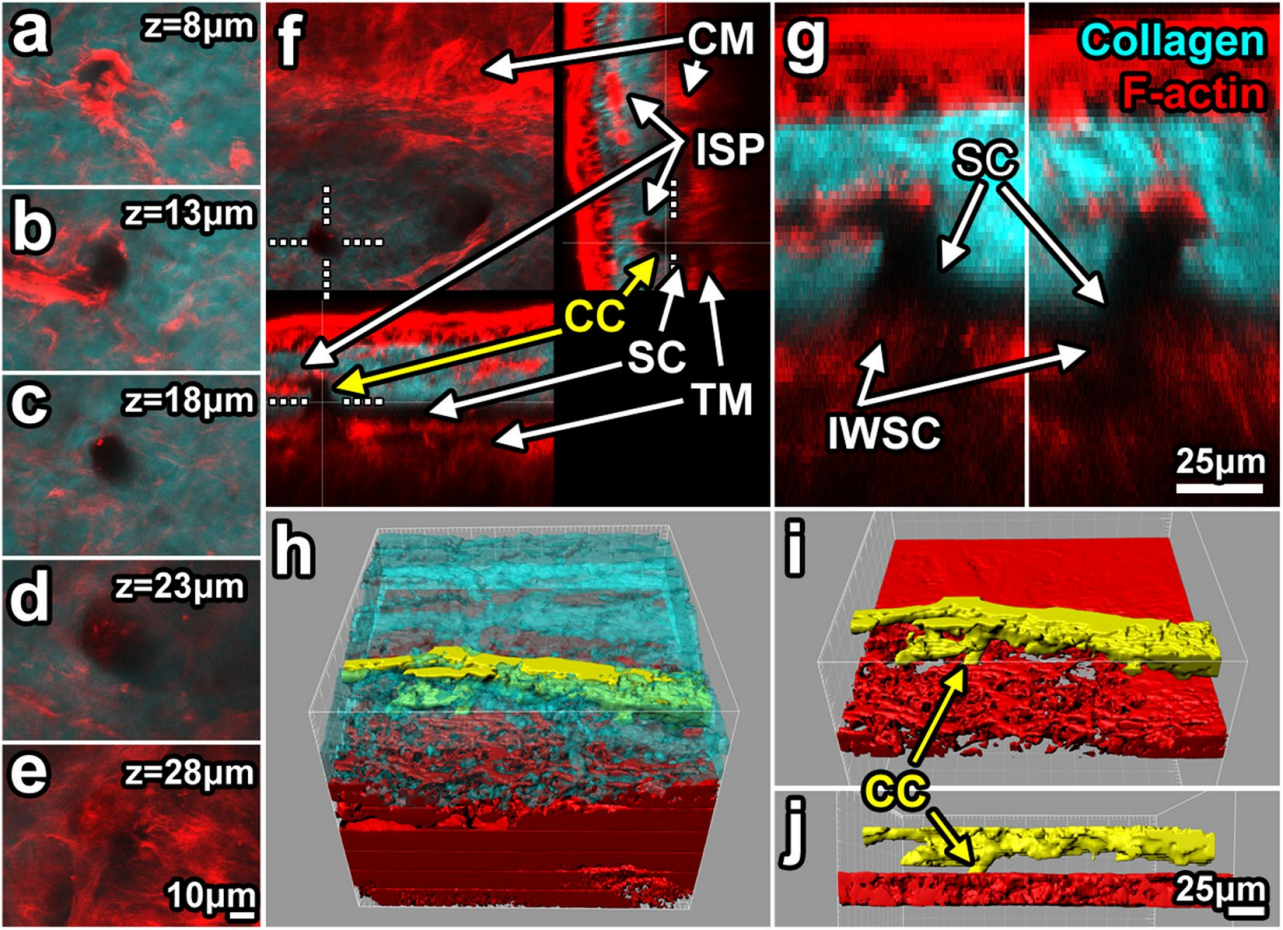
Detail of filamentous actin associated with intrascleral channels. (**a**–**e**) 2-photon serial optical sections through a collector channel (CC) show filamentous actin (F-actin; red) association with the wall of a CC (signal void in collagen second harmonic generation (SHG; cyan)). (**e**) CC ostium in the outer wall of Schlemm’s canal (OWSC; SC). (**f**,**g**) F-actin signal was relatively intense in the trabecular meshwork (TM)/inner wall of SC (IWSC) region. Orthogonal reconstruction showed CC ostium (*dotted line*) association with a classic right-angled CC (~17 μm diameter) connecting to an intrascleral plexus (ISP). (**g**) 3X magnification of the ostium shown in **f**. (**h**–**j**) 3D reconstruction from isosurface maps showed ISP association with CCs (yellow; from solid-modeling of SHG signal voids) within the sclera (cyan; **h**). Scleral collagen SHG was rendered transparent (**h**) to reveal associations in 3D tissue space. TM reconstruction (red) revealed a latticed mesh-like structure (**h**–**j**). *z:* tissue depth relative to the outer scleral surface.

Phalloidin-Alexa-568 labeling of C57BL/6 mouse eyes *ex vivo* showed prominent F-actin in the walls of ISP, CC and SC (Figs 7 and 8) in a pattern reflecting a multilayered cell organization (Fig. 7j,k). The wall distribution of F-actin labeling was similar to that seen of m-Tomato expression (Fig. 2), with both yielding a brighter and broader signal band than that expected of endothelium based on the diaphanous Prox1-GFP signal (Fig. 6j–k, m-n; arrowhead). F-actin-positive labeling was continuous throughout the walls of ISP, CC and SC, as seen in serial optical sections (Figs 7b–i; 8a–f) and orthogonal reconstructions (Figs 7a; 8f,g).

**Figure 9:**
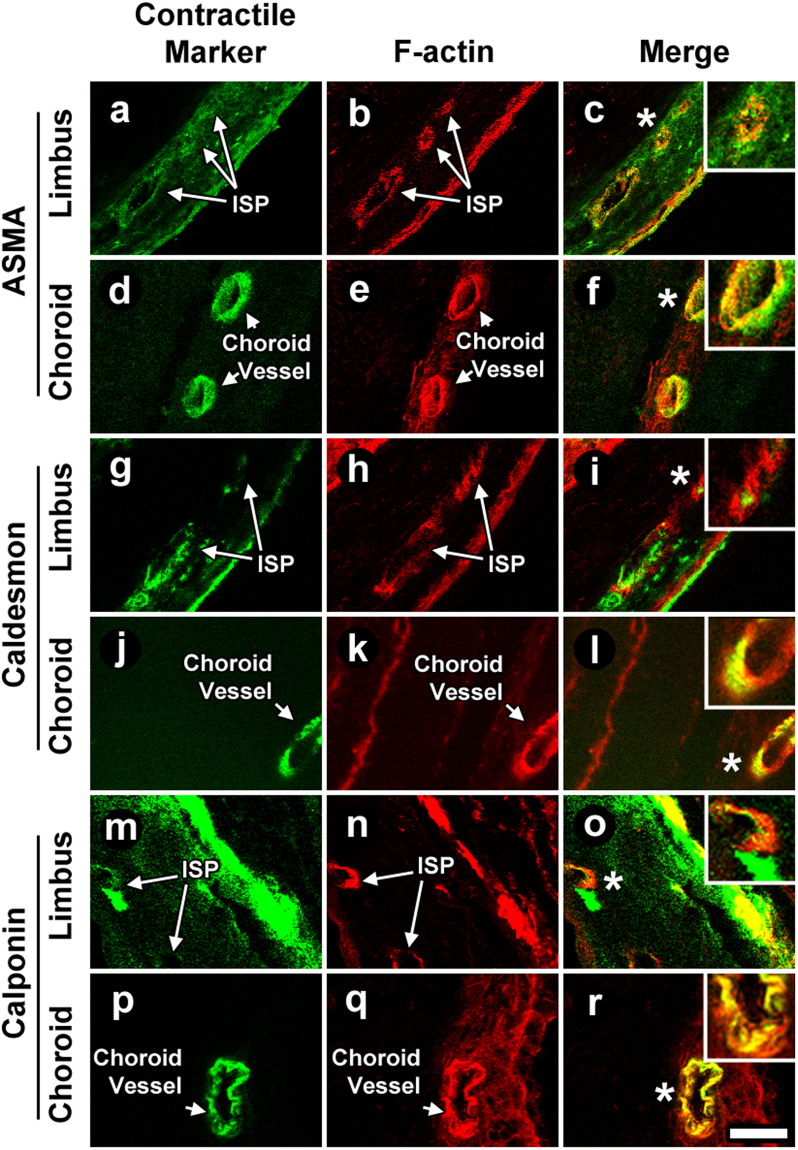
Contractility profile of the distal aqueous drainage tract compared to chordoial arteries. *C57BL/6* mouse eye frozen sections were labeled for filamentous action (F-actin) with (i) Alexa-568-conjugated phalloidin (**b,e,h,k,n,q**) and (ii) antibodies to alpha-smooth muscle actin (ASMA; **a,d**), caldesmon (**g,j**), or calponin (**m,p**). The labeling profile of ASMA, caldesmon and calponin was similar in distal aqueous drainage walls (intrascleral plexuses [ISP] shown; **a-c**, **g-i**, **m-o**) and choroidal arteries (**d-f**, **j-l**, **p-r**). F-actin co-localized with ASMA, caldesmon and calponin in these same locations (ISPs: **c,i,o**; choroidal arteries: **f,l,r**) *Insets*: 2X magnifications of regions indicated by asterisks. Bar = 25 μm.

We validated our *in situ* F-actin findings obtained by *en face* TPEF by comparing them with phalloidin-Alexa-568 labeling in immunohistochemistry (IHC) of radial histological sections of C57BL/6 mouse eyes. IHC findings mirrored those of TPEF wherein prominent F-actin labeling was seen in ISP walls (Fig. 9b,h,n). Furthermore, the pattern of F-actin labeling in ISP walls was similar to that seen in the walls of choroidal arteries (Fig. 9e,k,q).

We postulated that the positive F-actin labeling in ISP walls corresponded in large part to the location of a smooth muscle coat as would typically be seen in blood vessels such as arteries. The mouse eye tissues were antibody-labeled for classic smooth muscle epitopes of alpha smooth muscle actin (ASMA), calponin and caldesmon. These proteins are involved in smooth muscle contractility and expressed in the smooth muscle of CM^16^ and arterial walls^52–55^. IHC analysis showed that all three smooth muscle proteins localized to ISP walls (Fig. 9a,g,m) in a profile similar to that seen in choroidal arterial walls (Fig. 9d,j,p). All three smooth muscle epitopes of ASMA, calponin and caldesmon co-localized with F-actin in ISP walls (Fig. 9c,i,o). The pattern of smooth muscle epitope co-localization with F-actin mirrored that seen in choroidal artery walls (Fig. 9f,l,r).

**Figure 10:**
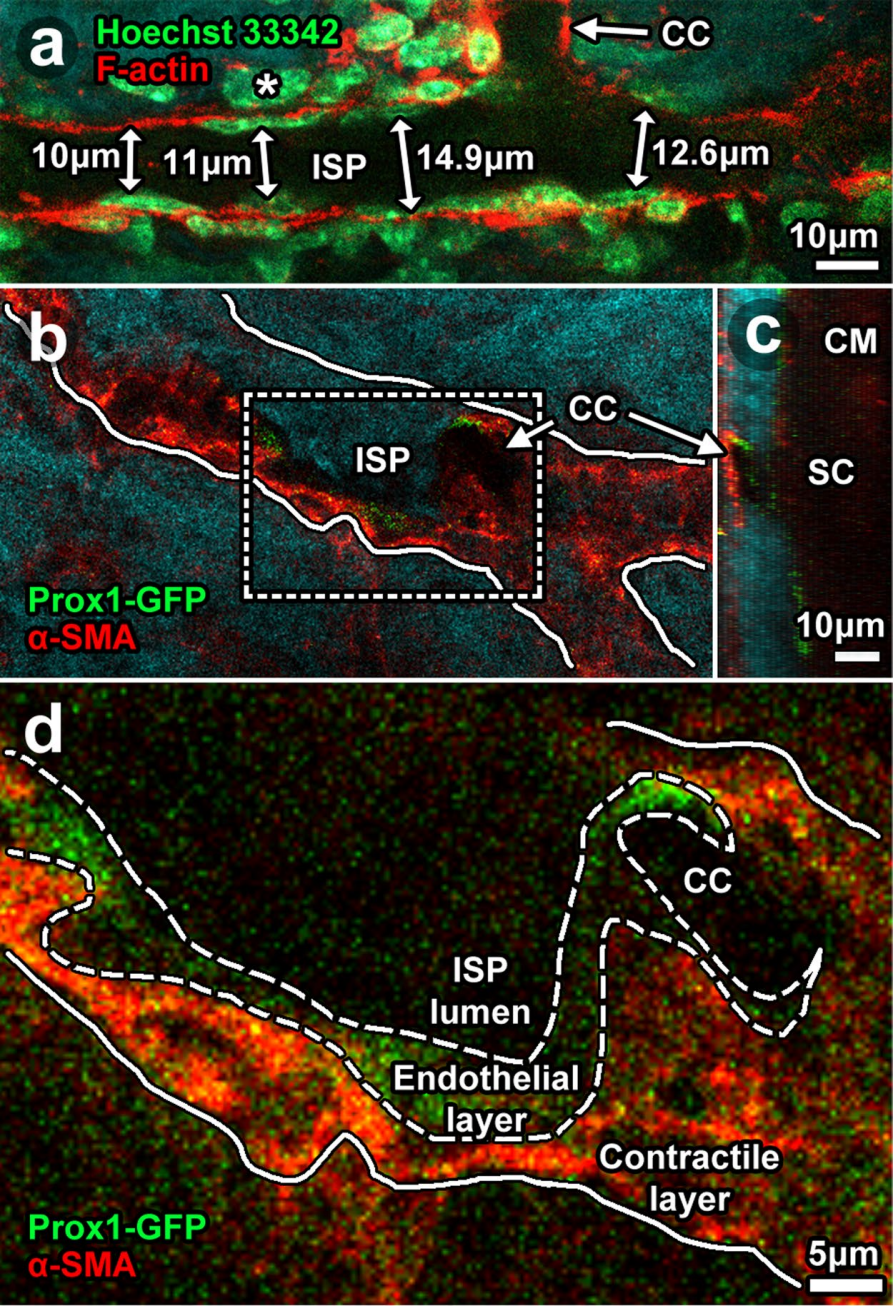
Cellular arrangement in distal aqueous drainage tract walls. 2-photon imaging *ex vivo* after labeling with: Hoechst 33342 for nuclei (**a**; *C57BL/6* mouse eyes); phalloidin-Alexa-568 for F-actin (**a**); and anti-alpha-smooth muscle actin antibodies (ASMA in *Prox1-GFP* mouse eyes; (**b**–**d**). (**a**) A thin cellular layer with flat nuclei lay next to the lumen (dark signal void) of an intrascleral plexus (ISP) connecting with a collector channel. This Prox1-GFP-positive layer (**b**, low magnification; (**c**), orthogonal reconstruction; (**d**), high magnification) was consistent with distal tract endothelium. A multilayered cellular layer external and adjacent to the endothelium had plump nuclei and expressed ASMA but not Prox1-GFP. This ASMA-positive layer was consistent with a contractile smooth muscle layer. It correlated with the ASMA-, calponin- and caldesmon-positive regions that prominently co-localized with F-actin labeling shown in Fig. 9. Double-sided arrows represent ISP lumen diameter measurements nearest the collector channel ostium (CCO).

We explored further the idea that cells with a smooth muscle identity form a layer external to endothelium in the distal aqueous drainage tract. High magnification TPEF analysis of C57BL/6 mouse eyes (Fig. 10) showed cells with flat nuclei (Hoechst 33342-positive) associated with thin F-actin-positive cytoplasm in an endothelial monolayer bordering the lumen of ISP (Fig. 10a). Immediately external to the endothelium were cells with plump nuclei and more diffuse F-actin-positive cytoplasm in a multilayer arrangement (Figs 7j,k; 10a). Location of the endothelial layer was consistent with a Prox1-positive layer bordering ISP and CC lumen of the Prox1-GFP mouse eyes (Fig. 10b–d). The plump nuclei in a multilayer arrangement immediately external to the endothelium (Fig. 10a) correlated with ASMA-positive labeling in a layer immediately external to the Prox1-positive region (Fig. 10b–d). The diameter of ISP lumen had a range of 9–15 μm (Fig. 10a).

### Profiling contractile markers in proximal and distal aqueous drainage tract

We then compared the DT profile of contractile markers with that of the more proximal aqueous drainage tissues of TM (conventional) and CM (unconventional) in which contractility is of functional importance.

**Figure 11:**
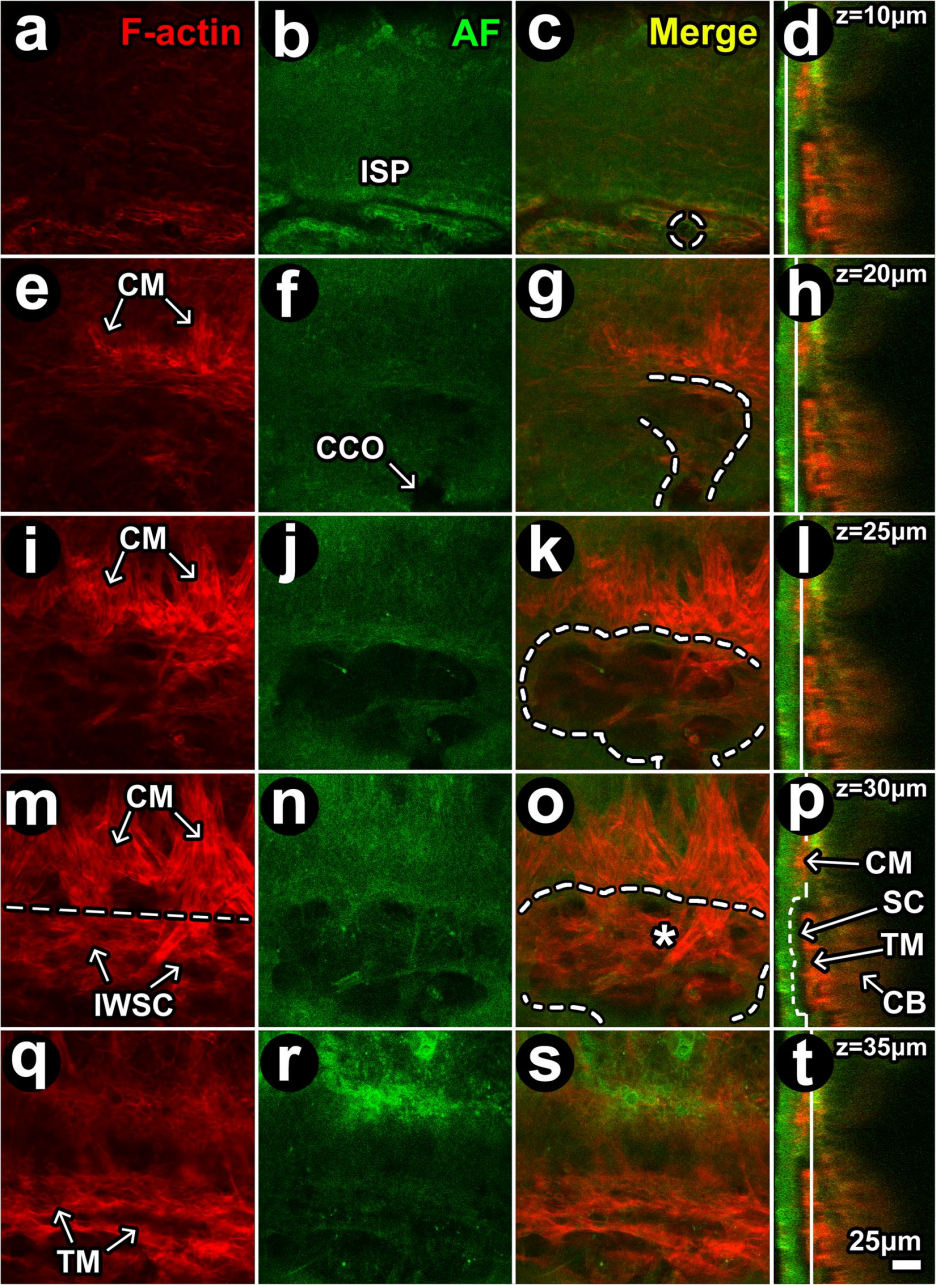
Filamentous actin in proximal and distal aqueous drainage tract. (**a**–**d**) at z = 10 μm (from the external scleral surface), autofluorescence (AF; green) signal voids (dark) revealed intrascleral plexuses (ISP) lined by filamentous actin (F-actin; red). *Dashed circle:* location of ostium more proximally in Schlemm’s canal (SC). (**e**–**h**) At z = 20 μm, F-actin-positive ciliary muscle (CM) fibers oriented perpendicular to ISP attach at a border region posterior to SC analogous to scleral spur. *Curved dashed line:* outlines SC lumen within a scleral depression (AF signal void). (**i**–**l**) At z = 25 μm, more proximal in SC lumen (**j**; dashed outline in **k**), F-actin-positive CM fibers were more prominent and crossed into the region of SC (within dashed outline) within the trabecular meshwork (TM)/inner wall of SC (SCIW) interface. (**m**–**p**) At z = 30 μm, vertically-oriented F-actin-positive CM fibers intermingle with cellular F-actin in the TM/IWSC interface region. Horizontal line places an imaginary border between CM and TM/IWSC regions (*asterisk*). The AF signal (**j,n**) revealed septae and loculation in SC. (**q**–**t**) At z = 35 μm, an F-actin-positive mesh-like labeling pattern was seen in the TM. Red: F-actin (**a,e,i,m,q**). Green: AF from structural extracellular matrix (**b,f,j,n,r**). Merged optical sections: (**c,g,k,o,s**) (red-green). Merged orthogonal reconstructions: (**d,h,l,p,t**) (red-green). Vertical white line in orthogonal image: z-location of optical section. Curved dashed lines: outline a lumen that continued from ISP to collector channel (CC; *dashed circle*) to CC opening (CCO) to SC.

Balb/c albino mice lacking pigment in the otherwise heavily pigmented TM and ciliary body were used to optimize imaging of the deeper proximal aqueous drainage structures. Scleral autofluorescence (AF), having its source in structural collagen and elastin^35,39,43^, was detected by trans-scleral *en face* TPEF imaging (Fig. 11). The AF signal revealed similar scleral features to those seen with SHG: AF signal voids revealed a pattern of ISP connecting with CC and SC (Fig. 11b,f,j,n,r vs. Figs 1b, 2, 7, 8, 10).; and AF signals revealed septae within SC so that the SC lumen appeared loculated (Fig. 11j,n).

Serial optical sections showing F-actin-positive signals (Fig. 11a,e,i,m,q) and merged F-actin and AF signals (Fig. 11c,g,k,o,s) revealed F-actin-positive CM fiber bundle extensions intermingling with F-actin-positive cells in the TM/IWSC region overlying SC.

In the TM (just deep to SC; Fig. 11m-t), F-actin-positive labeling and AF signals had a similar pattern. This indicated coincidence of F-actin-positive cells (Fig. 11s) with trabecular-like beams (Fig. 11r) surrounding tissue pores (AF signal voids; Fig. 11r-s). This beam-pore arrangement was reminiscent of the human TM.

**Figure 12:**
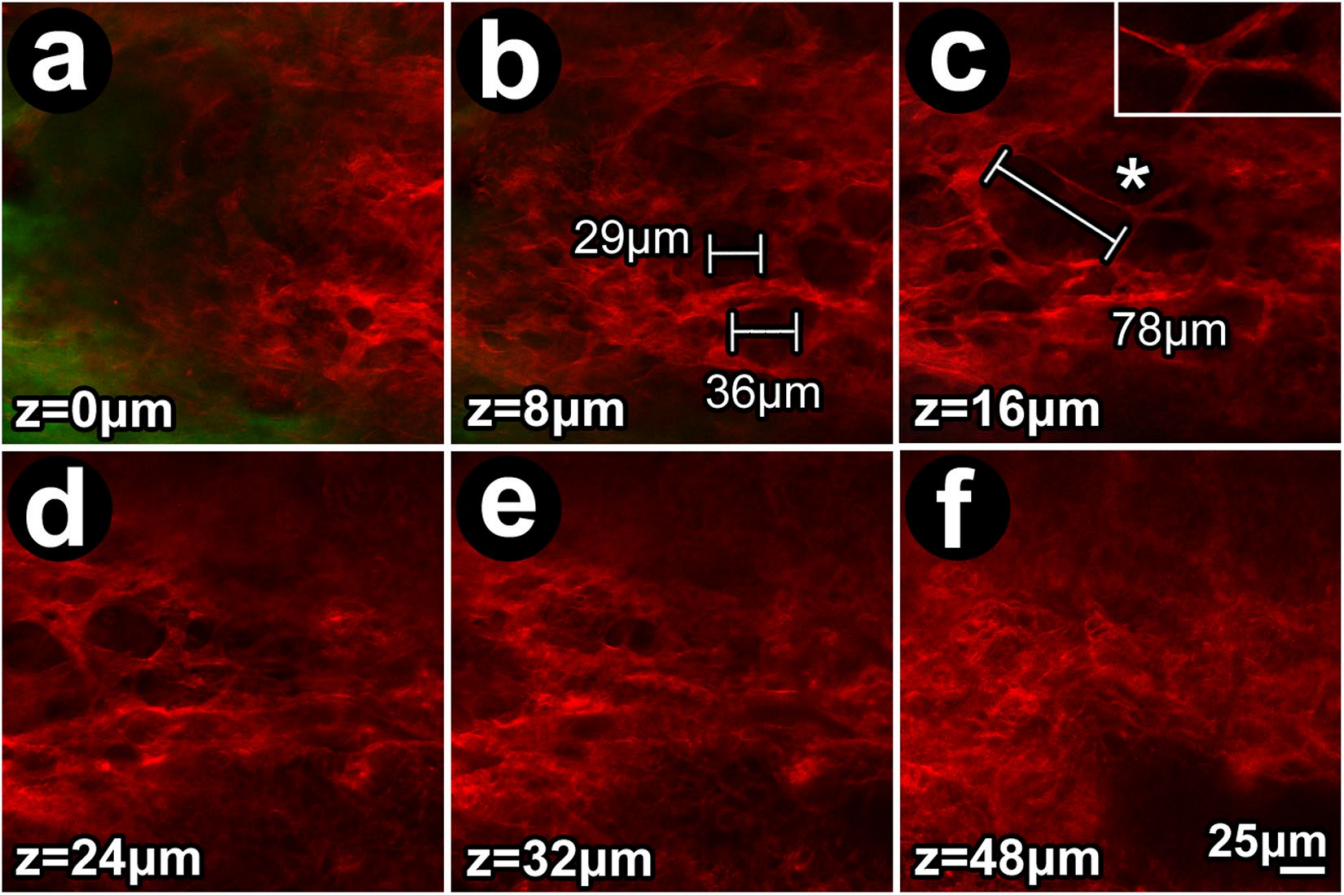
Filamentous actin distribution in mouse trabecular meshwork. (**a**–**c**) Pore-like gaps, similar to those found in human TM, were evident in the mouse TM (**a,b**, and **c** inset). At z = 8 μm internal to IWSC, gaps had diameters of 20–40 μm (**b**), but at z = 16 μm, further internal to IWSC, gap diameters exceeded 40 μm (**c**), reminiscent of pore size variation with depth in the human trabecular meshwork. (**d**–**f**) Further internally, the mouse TM interfaced with ciliary muscle (**d,e**) in which the pattern of large pore-like gaps was absent. Green: autofluorescence from structural extracellular matrix (**a**; predominantly sclera). *Insets:* 2X magnifications of regions indicated by asterisks.

The notion that F-actin tissue distribution is similar in mouse and human TM was explored further (Fig. 12). Similar to human TM, gaps between F-actin-positive labeling in mouse TM increased in size with tissue depth (i.e., further internal from SC; Fig. 12a–c) until a transitional region consistent with CM was reached just deep to the TM (at tissue depths seen in Fig. 12d–f).

**Figure 13:**
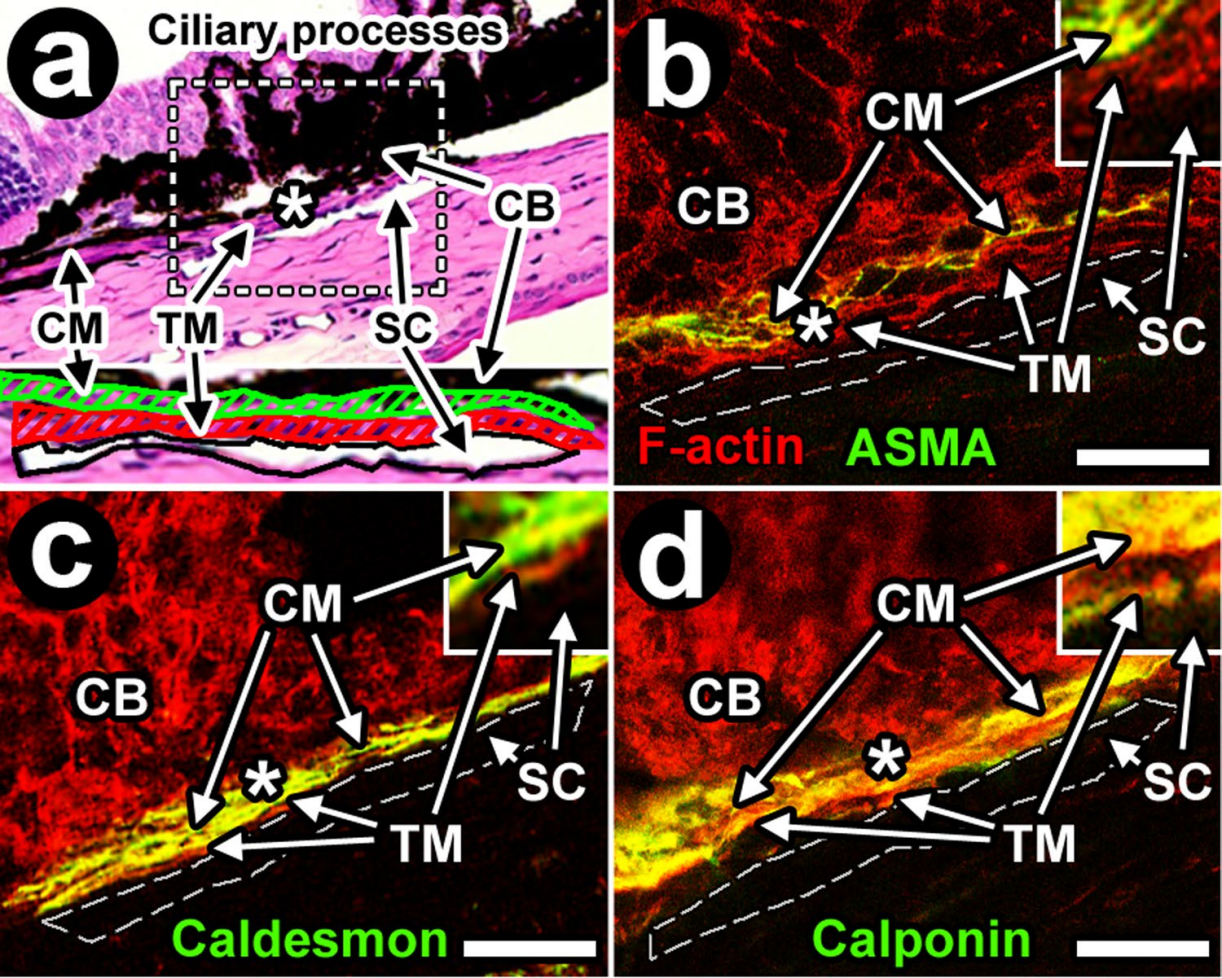
Ciliary muscle but not trabecular meshwork displays smooth muscle features resembling those of distal aqueous drainage tract wall. Immunohistochemistry (IHC) showing regions where filamentous actin (F-actin; red) and smooth muscle epitopes (alpha smooth muscle actin (ASMA; **b**), caldesmon (**c**) or calponin (**d**; all green; see *insets*)) co-localize (yellow; **b**–**d**). Co-localization between F-actin and smooth muscle epitopes is seen in ciliary muscle (CM; yellow), which is smooth muscle, but not in trabecular meshwork (TM; *thin red band* next to Schlemm’s canal (SC)). (**a**) H&E staining of a *C57BL/6* mouse eye illustrates the orientation of proximal aqueous drainage tissue^16^. *Dashed box:* iridocorneal angle drainage tissue region that was the focus of subsequent IHC images (**b**–**d**; *Balb/c*). Lack of pigment in albino *Balb/c* mice allowed unbiased analysis of fluorescence labeling across the otherwise pigmented drainage tissues. (**b**–**d**) Dashed line: outline of SC. F-actin was present in the TM (red) and CM, but F-actin co-localization (yellow) with ASMA (**b**), caldesmon (**c**) and calponin (**d**) was a prominent feature of CM but not TM. *Insets:* 3X magnification of regions indicated by asterisks illustrating prominent F-actin co-localization with smooth muscle epitopes in CM (yellow) but not TM (red). Bar = 20 μm.

We explored the possibility that F-actin-positive cells in mouse TM and CM expressed classic smooth muscle epitopes of ASMA, calponin and caldesmon, which if present would indicate a smooth muscle identity (Fig. 13; Balb/c)^16^. In histological sections, the mouse TM is a cellular layer just adjacent and internal to SC, while the CM lies adjacent and internal to TM as a muscle layer sandwiched between the TM and pigmented ciliary body^16^. Our IHC analysis showed positive labeling of ASMA, caldesmon and calponin in the CM but not TM. ASMA, caldesmon and calponin labeling also co-localized with F-actin in the CM but not TM. The CM labeling profile was reproducible for all smooth muscle markers tested (i.e., ASMA, caldesmon and calponin; Fig. 12b,c) and mirrored the labeling profile and pattern of co-localization with F-actin for the same markers in the DT walls and choroidal arteries (Fig. 9). Neither smooth muscle markers nor their co-localization with F-actin was seen in the TM, a contractile aqueous drainage tissue that is not considered to be smooth muscle.

## Discussion

We have assembled tools for tissue-based and *in vivo* studies of the mouse to address goals to: (1) identify and verify the DT in deep tissue imaging; (2) digitally map the 3-dimensionally complex DT and its relationships; (3) better characterize the unique endothelial molecular signature of the DT relative to lymphatic and blood vessels; (4) explore the possibility that the DT has capacity to contract and carries a smooth muscle identity mimicking that of blood vessels; and (5) compare the DT profile of contractile markers with that of proximal drainage tissues of the TM and CM. We exploited transgenic reporter mice and multi-modality trans-scleral 2 P imaging incorporating TPEF and SHG. Endogenous and exogenous fluorophores were visualized at a subcellular-level in the DT. We were able to study the DT in a way that preserved its 3D organization and connection to the systemic vasculature, which is important functionally and could provide a context for better understanding its specific physiology and molecular regulation.

The first step we took to verify that structures under study were part of the DT was to trace them to their origin in SC. Lumen of the DT were evident as voids in the scleral SHG or AF signal. These voids representing lumen could be traced and mapped using software reconstruction techniques we have previously described^40,43–45^. The primate SC connects with downstream vessels via CC at intervals along the length of SC. It has been assumed that mice also have such an organization but this had not directly been shown prior to this study. Downstream from CC, ISP mapped as a large interconnected endothelial-lined collector system within the mid to superficial sclera. ISP connected via CC to its deep scleral origins in SC. CC varied in shape, size and orientation in their intrascleral course between ISP and SC.

**Figure 14:**
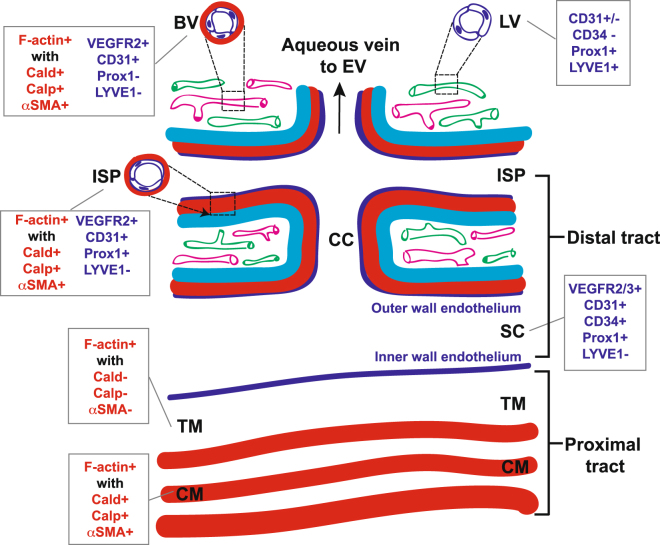
Mouse distal aqueous drainage tract endothelium and contractile marker profiles, illustrated with reference to the more proximal trabecular meshwork and ciliary muscle and based on tissue-based observations. BV: Blood vessel (pink); LV: Lymphatic vessel (green); ISP: Intrascleral plexus; CC: Collector channel; SC: Schlemm’s Canal; TM: Trabecular meshwork; CM: Ciliary muscle; F-actin: Filamentous actin; Cald: Caldesmon; Calp: Calponin; αSMA: Alpha-smooth muscle actin. Red: contractile layer; dark blue: endothelium; cyan: sclera. Red text: Contractile markers; blue text: endothelial markers.

A second step in identifying the DT was to detect endothelial expression of Prox1. Prox1 co-localization with LYVE-1 (for true lymphatics) and/or global endothelial markers (e.g., CD31) reveals differential selectivity for true lymphatic vessels and blood vessels and was used to help distinguish the different vascular systems by their respective endothelial signatures at the limbus where imaging was performed. It was based on an understanding that Prox1 is expressed in both the DT and lymphatic vessels but not in the blood vessels; CD31 is strongly expressed in the DT and blood vessels but not in lymphatics; and LYVE-1 is expressed in lymphatics but not DT or blood vessels^26,27^. Our findings are summarized in Fig. 14.

Prox1-expressing endothelium bordered lumen of the DT^20–23^. In SC, Prox1 expression was more prominent in the IWSC than in the OWSC, as has been reported^24^. Prox1 endothelial expression was also present in CC and ISP, which has been suggested^25^ but not widely recognized. Prox1-GFP expression in CC and ISP appeared as a diaphanous signal bordering the lumen (e.g., Fig. 6j,k,m,n) that could also appear patchy depending on the plane of capture (Fig. 5g,h). Perhaps this subtlety explains why endothelial Prox1 expression in more distal parts of the DT is not better recognized. Our 2 P imaging had deeper tissue penetration than conventional 1 P imaging that has previously been used to study this system. The 2 P imaging permitted more sensitive detection of fluorophores deep in tissue. Our finding of widespread Prox1 expression in the DT contributes to a fuller characterization of the system. It is also important because Prox1 has a putative role in mechanical biosensing^25^ that is relevant to the regulation of IOP and glaucoma.

F-actin is the polymerized, contractile form of actin. F-actin-positive labeling was used to generally localize cells in a contracted state within the tissues. The profile of F-actin-positive labeling in the DT of WT mice (Fig. 7) was similar to that of the cellular membrane-targeted td-Tomato signal in *mT/GFP* mice (Fig. 2). Both yielded a broader and more prominent signal bordering the lumen compared with the diaphanous Prox1-GFP-positive endothelial signal in *Prox1-GFP* mice (Fig. 6m,n). This led us to postulate that the F-actin signal encompassed a further cellular complex external to the endothelial monolayer. Higher magnification images indicating a multi-layered configuration of F-actin-positive cells external to endothelium (Figs 7j,k, 10) supported this notion. That F-actin primarily co-localized with mesenchymal markers of ASMA, caldesmon and calponin (Fig. 9) - which is not expected in true endothelium - was also in line with this.

We propose that the F-actin signal that co-localized with ASMA, caldesmon and calponin in DT walls originated in mural contractile cells with a smooth muscle identity. These contractile cells reside in a DT wall region analogous to the tunica media smooth muscle wall layer of blood vessels. A mesenchymal identity is not expected in true endothelium. That the labeling profiles of ASMA, caldesmon and calponin in DT walls were similar to those seen in arterial smooth muscle walls and that the proteins also co-localized with F-actin lends further credence to this idea. Furthermore, the smooth muscle marker labeling profile in the DT wall mirrored that of CM, which itself is smooth muscle, as illustrated in Fig. 14. ASMA association with human SC and CC has been reported before^11,12^.

We measured mouse CC ostia to have lumen diameters in the scale of 10 μm, which is comparable to lumen diameters of human metarterioles (5–10 μm)^56^. Human metarterioles have a three-layered stratification: (1) tunica intima featuring attenuated endothelial cells on a basement lamina with scarce elastic and collagen fibers; (2) tunica media of smooth muscle cell layers; and (3) tunica externa of collagen mixed with autonomic nerve fibers, fibroblasts, histiocytes, and mast cells. The mouse DT appears to share features of this wall organization. We postulate that the mouse analogue of the tunica externa is the sclera itself, or maybe a fine collagenous condensation of the sclera that was imperceptible by our SHG imaging. That the DT and arteries have a common wall design likely reflects a common need of different vascular systems to constrict to modulate drainage resistance and intermittently distend and recoil to accommodate pulsatile flow.

The extent to which the intrascleral DT is able to distend within the relatively rigid eye coat is unclear. If the DT has significant compliance it would perhaps align it more with a venous than arterial analogue, providing a parallel paradigm for conceptualizing the tract’s function. Alternatively, as the DT connects to the episcleral veins, one could conceive of an episcleral venous compliance system directly downstream from the DT in which the DT serves primarily as a resistor. Compared with arteries, veins have a less prominent contractile layer and more prominent lumen, a design that supports compliance and responses to changes in hemodynamic pressure. Venous features are not necessarily static, however, as they may adapt over time to dynamic forces. A classic example is the clinical observation of arterial features developing in episcleral veins in response to prolonged elevated episcleral venous pressure - so called ‘arterialization of veins’. These questions conceptualizing the DT as vascular analogues could stimulate new ways to understand IOP regulation and seem worthy of further attention.

We found that the mouse TM showed prominent F-actin labeling but lacked the smooth muscle phenotype seen in the DT, CM and choroidal artery walls. While the TM is regarded to be contractile^44,57,58^ it seems to be a different type of contractile tissue to that of the CM^16^ or DT. The TM typically does not exhibit smooth muscle features except perhaps in a context of presumed mesenchymal transition associated with ASMA upregulation and a heightened myofibroblastic contractile state^36,59–62^. CM fibers inserting in the region of the TM could themselves contribute to contractile forces exerted on and within the TM. CM elements intermingling within the TM may explain why the TM, even in a physiological state, could appear to have histochemical mesenchymal features reminiscent of muscle. It may be that resident TM cells displaying non-muscle actomyosin contractility play roles in physically organizing the extracellular matrix to counter extraneous muscular and hydrodynamic forces exerted on the tissue. Somehow the system must find balance. This is necessary to promote outflow homeostasis by the conventional (TM and DT) and unconventional (CM) tracts, which together determine IOP^57,58^. By what means homeostasis is orchestrated by the different drainage tissue components and what are sources of homeostatic malfunction leading to disease remain important questions for future study.

Our data indicates that the walls of DT channels extending from SC to ISP maintain certain common features across their different parts, namely Prox1-expressing endothelium adjacent to an external contractile cellular compartment with smooth muscle identity. Clues to this arrangement were evident in *in vivo* and *ex vivo* imaging, with the techniques cross-validating each other. The agreement suggests that 2 P imaging *ex vivo* may suffice to capture data representative of the *in vivo* context in appropriate cases. *Ex vivo* imaging is simpler to perform than *in vivo* imaging, which is subject to additional challenges such as motion artifact. The limitation of *ex vivo* imaging, however, is that the aqueous physiological outflow dynamic is absent.

We speculate that the DT is not merely a system of passive channels within the sclera. Rather it has capacity to contract, with potential to modulate caliber and outflow resistance in analogous fashion to the modulation of vascular resistance by blood vessel tone. The interplay of systemic vascular resistance and compliance plays critical roles in maintaining hemodynamic stability and it is not inconceivable that the DT plays comparable roles in IOP homeostasis. The existence of a pulsatile component to aqueous outflow under the influence of cardiovascular hemodynamics^63–68^ further argues for a DT with capacity to dynamically modulate tone and flow resistance as blood vessels do. The tissue-based and live mouse approaches we have described provide useful options for dynamic *in vivo* studies that are appropriate next steps in characterizing the specific cell biology, physiology and pharmacology of the DT.

## Materials and Methods

### Reagents

The following reagents were used: Fluorescence tag-conjugated phalloidin and ProLong Gold Anti-fade with 4′,6-diamidino-2-phenylindole (DAPI; both from Life Technologies, Grand Island, NY, USA); optimum cutting temperature (OCT) compound (Sakura Finetek, Tissue-Tek, Torrance, CA, USA); bovine serum albumin (BSA), paraformaldehyde, and triton X-100 (Sigma (St. Louis, MO, USA); low glucose DME, L-glutamine, amphotericin B, and DMSO (to dissolve calcein AM; Mediatech, Washington, D.C., USA); penicillin/streptomycin (Norris Comprehensive Cancer Center Cell Culture Core, Los Angeles, CA, USA); and serum-free media comprising low glucose DME, streptomycin, penicillin, gentamicin and L-glutamine.

### Antibodies and fluorescent dyes

Alexa Fluor^®^ 488, 568, or 633 conjugated secondary antibodies were purchased from Life Technologies. Cy5-conjugated anti-rabbit secondary IgG was purchased from Jackson ImmunoResearch Laboratories (West Grove, PA, USA). Rabbit or goat anti-ASMA (Cat # ab5694 or ab21027, 1:100 dilution), rabbit anti-caldesmon (E89, Cat # ab32330, 1:200 dilution), and rabbit anti-calponin (Clone EP798Y, Cat# ab5694, 1:100 dilution) were purchased from Abcam (Cambridge, MA, USA). Rabbit anti-vascular endothelial growth factor receptor 2 (VEGFR2) was purchased from R&D Systems (Minneapolis, MN, USA) and rabbit anti-lymphatic vessel endothelial receptor 1 (LYVE-1) from AngioBio (San Diego, CA, USA), and anti-CD31 from Dako (Glostrup, Denmark). Normal goat, rabbit, and mouse IgG isotypes were purchased from Santa Cruz Biotechnology, Inc. (Santa Cruz, CA, USA). DAPI was purchased from Vector Labs (Burlingame, CA, USA). Enucleated mouse eyes were incubated with 100 μM siGLO transfection indicator and Accell siRNA delivery media (Dharmacon/GE Healthcare, Wauwatosa, WI, USA) for 24 h at 37 °C and 8% CO_2_. Trans-scleral 2 P microscopy was used to capture images of intact siGLO labeled-eyes without fixation, dissection, or post-processing. Application of the different fluorophores is summarized in Table 1.

**Table 1:** Fluorescence markers.

Fluorpohore	Acronym	Represents	Marker for:
Endogenous	GFP	Green fluorescence protein	Cytoplasm
	Prox1-GFP	Prospero homeobox protein 1	Endothelium of distal tract or lymphatics
	mTomato	membrane-localized tandem dimeric red fluorescent protein (td-Tomato)	Plasma membrane
	VE-cadherin-td-Tomato	Vascular endothelium cadherin	General endothelial marker
Epifluorescence	CD31/PECAM1	Cluster of differentiation 31/Platelet endothelial cell adhesion molecule	General endothelial marker
	F-actin	Filamentous actin	Contractile cytoskeleton
	Hoechst 33342	Hoechst 33342	Nucleic acid stain
	LYVE-1	Lymphatic vessel endothelial hyaluronic acid receptor 1	Lymphatic endothelium
	siGLO	Fluoroscein amidite-labeled RISC independent transfection control	Nucleus
	VEGFR2	Vascular endothelial growth factor receptor 2 precursor	General endothelial marker
Autofluorescence	AF	Autofluorescence of elastin & fibrillar collagens (e.g. types I and III)	Elastin and fibrillar collagen

### Animal husbandry and anesthesia

Experiments complied with the ARVO Statement for Use of Animals in Ophthalmic and Vision Research. Approval was obtained from the Institutional Animal Care and Use Committees (IACUC) at the University of Southern California and University of California, Los Angeles.

Mice were housed and raised in air-filtered clear cages in a 12-hour light/dark cycle environment and fed ad libitum, as previously described^45^. A ketamine-xylazine-acepromazine mix was used for intraperitoneal anesthesia^45^. Prior to imaging, topical proparacaine hydrochloride ophthalmic solution (0.5%, Akorn, Inc, Buffalo Grove, IL, USA) was dropped on the cornea. Body temperature was maintained during imaging by resting mice on a warming platform or under a heating blanket (Homeothermic Blanket Systems, Harvard Bioscience, Inc., Holliston, MA, USA).

The following mice were used: (a) *C57BL/6* and *Balb/c* (Charles River Laboratories (Wilmington, MA, USA; aged 3–4 months). (b) GFP/membrane-targeted td-Tomato (*GFP/mT*) hybrid mice *for in vivo* and *ex vivo* studies, as described previously^45^. They were a generous gift from Dr. Roberto Weigert (NIDCR), and had been derived from the *GFP/mT* mouse by crossing *Friend Virus B-Type* transgenic mice expressing EGFP^69^ with *C57BL/6* mice expressing the membrane-targeted td-Tomato protein (m-Tomato; Jackson Laboratory, Bar Harbor, ME)^70^. A chicken β-actin promoter and cytomegalovirus enhancer drove transgene expression^71^. (c) *Prox1-GFP* ^28^, Prox1-td-Tomato (*Tg*(*Prox1-td-Tomato*)*TA76Gsat/Mmucd*), and *Cdh5(PAC)-CreERT2/Rosa26-td-Tomato/Prox1-EGFP (Prox1/VE-Cadherin)*, all generous gifts from Dr. Young Hong (University of Southern California), were used for *ex vivo* studies.

The *Cdh5*(*PAC*)-*CreERT2*/*Rosa26-td-Tomato*/*Prox1-EGFP* (Prox1-GFP/VE-Cadherin-td-Tomato) mouse was a *Prox1-GFP* mouse with inducible expression of td-Tomato fluorescence-tagged VE-cadherin (Chd5). It was generated by crossing *Cdh5*(*PAC*)*-CreERT2* ^72^ (provided by Dr. Ralf Adams, University of Münster, Germany) with *B6.Cg-Gt(ROSA)26Sortm14(CAG-td-Tomato)Hze/J* (Rosa26-td-Tomato; The Jackson Laboratory)^46^ and, ultimately, with Prox1-EGFP.

VE-cadherin-td-Tomato expression was induced by tamoxifen treatment over 48 h. Briefly, tamoxifen was dissolved in DMSO to a concentration of 20 mg/mL and further diluted 1:2 with sunflower oil. 1 mg of tamoxifen was injected intraperitoneally twice over a 24 h period.

### Two-photon imaging of *ex vivo* eyes

Whole enucleated mouse eyes, either fresh or fixed with 4% PFA and labeled for F-actin were imaged by 2 P microscopy as previously described^45^ (Leica TCS SP5 AOBS MP (Leica Microsystems, Heidelberg, Germany) or Zeiss 710NLO (Carl Zeiss AG, Oberkochen, Germany systems with multiphoton laser (Coherent, Santa Clara, CA, USA)).

Eyes were placed on a glass bottom dish (P35G-1.5-14-C; MatTek Corporation, Ashland, MA, USA), along with a drop of PBS, with the axis of the optic nerve and central cornea perpendicular to the direction of the beam. A 63X/1.3NA glycerol objective was used to focus an 850 nm wavelength laser to excite AF and Phalloidin-Alexa-568 fluorescence, as previously described^45^. The following multiphoton bandpass filters were used to collect endogenous or exogenously-labeled TPEF signals: 415–435 nm (for collagen SHG), 500–550 nm (for elastin AF and green fluorescence), and 590–680 nm (for red fluorescence). The location of SC and the TM (just deep to SC) was identified by the scleral collagen SHG void characteristic of SC lumen^34–37,43–45^. Some imaging was performed in Balb/c mice as their non-pigmented eyes allowed for relatively unimpeded trans-scleral views and higher quality images of deeper tissue regions such as ciliary muscle^16^ as previously described^16^. Table 2 summarizes imaging modalities used.

**Table 2:** Imaging modalities.

Modality	Acronym	Color	Example	Represents
1. Two-photon excitation fluorescence:	TPEF			
a. Endogenous fluorophores		Red	td-Tomato	Plasma membrane
			VE-cadherin-td-Tomato	Endothelium
		Green	GFP	Cytoplasm
			Prox1-GFP	Endothelium of distal tract or lymphatics
b. Epifluorescence		Red	CD31 (aka PECAM1)	Endothelium
			F-actin (Alexa- 568-Phalloidin)	Contractile cytoskeleton
			LYVE-1	Lymphatic endothelium
			VEGFR2	Endothelium
c. Intravital fluorescence		Green	Hoechst 33342	Nucleus
			siGLO	Nucleus
d. Autofluorescence	TPEF AF	Green	Elastin and fibrillar collagen	Elastin and fibrillar collagen (e.g. types I and III)
2. Second harmonic generation	SHG	Cyan	Fibrillar collagen	Fibrillar collagen (e.g. types I and III) ex: 850 nm; em: 425 nm
3. Isosurface mapping				
a. Signal		Cyan	Collagen SHG	Fibrillar collagen (e.g. types I and III)
		Green	siGLO	Trabecular meshwork
		Red	VE-cadherin-td-Tomato	Blood vessel endothelium
b. Signal voids		Yellow	Schlemm’s canal lumen	Distal drainage tract
			Collector channel lumen	Distal drainage tract
			Intrascleral plexus lumen	Distal drainage tract
4. Immunohistochemistry	IHC	Red	F-actin	Contractility marker
(by one-photon, confocal microscopy)		Green	alpha-smooth muscle actin	Contractility marker with smooth muscle identity
			Caldemson	Contractility marker with smooth muscle identity
			Calponin	Contractility marker with smooth muscle identity

Images (1024 × 1024 pixels and 16-bit grayscale resolution) were captured in LAS AF (Leica) and analyzed with Volocity 5.4.1 (PerkinElmer; Waltham, MA, USA), LAS AF Lite 2.2.1 (Leica), Imaris 7.3.0 (Bitplane; Zurich, Switzerland) or Image J (NIH; Bethesda, MD, USA), and edited for publication with Photoshop CS5 (Adobe), as previously described^45^.

### Two-photon imaging of live mice

An objective inverter (LSM Technologies)^73^ was used to image live mice following anesthesia as described previously^45^. Contact between the objective and the mouse eye was mediated with an optical coupling gel (0.5% Carbomer 940, 300 mM D-sorbitol, adjusted to pH 7.3 with triethanolamine; from Snowdrift farm, Tucson, AZ, USA).

A modified IX81 inverted 2 P confocal microscope (Olympus, Melville, NY, USA), coupled to a tunable Ti:Sapphire femtosecond laser (Chameleon Ultra II; Coherent) was used to image the live mouse eyes. The following bandpass filters were used to collect TPEF signals: 400–480 nm (for Hoechst fluorescence and collagen SHG), 505–560 nm (for FITC, Alexa-488, GFP/EGFP), and 590–650 nm (for Texas red and td-Tomato). All live mouse images and movies were acquired using a UPLSAPO 60X NA 1.2 water immersion objective in 2 P configuration^74^, as previously described^45^.

### Volumetric 3-dimensional (3D) tissue reconstructions

Imaris was used to generate “isosurface volumes” to recreate polygonal, volumetric renderings of tissue signals such as SHG, SHG signal voids, or endogenous and exogenously-labeled fluorescence markers (eg., AF, F-actin-phalloidin-Alexa568, Prox1-GFP, siGLO) as previously described^36,43–45^ and summarized in Table 2. Predetermined thresholds for size and fluorescence intensity were used to set minimum value requirements for volume rendering. Predetermined optimum threshold values^40,43^ were voxels of 0.24 × 0.24 × 1 μm in the X, Y, Z axes respectively. Where fluorescence dynamic range (especially of deep structures) was narrow relative to the dynamic range of shallower regions, reconstructions were cropped to a maximum optical slice thickness of 25 μm in the z-axis prior to isosurface volume rendering.

### Volumetric 3D reconstructions from signal voids

Imaris was used to reconstruct the SHG signal into “isosurface volumes” with the following parameters: 2 μm surface grain size; 1.803 μm largest sphere diameter; 3,600 minimum fluorescence intensity cutoff; and 10 μm minimum voxel size, as previously described^45^. The SHG isosurface volume was subtracted from a generic signal mask (all voxels of generic z-stack set to 20,000), leaving a negative SHG impression representing SC and ISP lumen. Isosurface volumes of SC lumen and ISP channel voids were reconstructed using the following parameters: 0.481 μm surface grain size; 1.803 μm largest sphere diameter; automatic (default) fluorescence intensity minimum cutoff value; and 10 μm minimum voxel size.

### Intrascleral plexus lumen diameter measurements

ISP cross-sectional diameters were measured at intervals of 25 microns from the center of the CC ostium. Measurements were obtained at each interval for every 2D frame of a z-stack containing a portion of the ISP lumen. The largest diameter measured among all frames at that interval was selected to represent the diameter of the ISP at that interval.

### Immunohistochemistry

7 µm-thickness frozen sections of enucleated eyes were fixed, permeabilized, and blocked in 4% PFA and blocking solution (5% BSA/0.3% Triton X-100) for 1 h at room temperature (RT), then incubated overnight at 4 °C with combinations of primary antibodies (single, dual, or triple) in the blocking solution. The sections were further incubated for 1 h at RT with a combination of secondary antibodies (Alexa Fluor^®^ 488, 568, and 633-conjugated anti-rabbit, mouse, and/or goat antibodies) and then mounted using ProLong Gold Anti-fade reagent with DAPI. To visualize polymerized actin, secondary incubation of slides included Alexa-568-phalloidin along with the appropriate combination of secondary antibodies. Negative, non-specific labeling was established with normal IgG isotypes. Sections were analyzed with a Leica SP5 high-speed spectral confocal laser-scanning microscope or Zeiss LSM 710NLO confocal microscope. Immunofluorescence staining for single or double contractile markers was performed in randomly selected slides (4–5 slides per each eye) containing 4 sections per slide.

Hematoxylin and Eosin (H&E) staining in paraffin sections of *C57BL/6* mouse eyes was performed as described previously^16^.

### Whole-mount immunofluorescence staining

Enucleated eyes were fixed with 4% PFA/PBS at 4 °C for 2 h prior to microsurgically isolating the whole cornea (i.e., including the anterior segment) from the posterior segment and lens. Whole corneas (and attached anterior segments) were washed 3X with PBS for 5 min then incubated with rabbit polyclonal anti-LYVE-1. After several washes the samples were incubated for 4 h at RT with Cy5-conjugated anti-rabbit antibody. Specimens were mounted using VectaShield mounting medium with DAPI (Vector Labs, Burlingame, CA, USA). Whole mount immunofluorescence images were captured using a Leica M165 fluorescent stereomicroscope (Leica).

### Data Availability

The datasets generated dGlaucuring and/or analysed during the current study are available from the corresponding author on reasonable request.
